# Production of highly and broad-range specific monoclonal antibodies against hemagglutinin of H5-subtype avian influenza viruses and their differentiation by mass spectrometry

**DOI:** 10.1186/s12985-017-0886-2

**Published:** 2018-01-15

**Authors:** Violetta Sączyńska, Anna Bierczyńska-Krzysik, Violetta Cecuda-Adamczewska, Piotr Baran, Anna Porębska, Katarzyna Florys, Marcin Zieliński, Grażyna Płucienniczak

**Affiliations:** Institute of Biotechnology and Antibiotics, Starościńska 5 Street, 02-516 Warsaw, Poland

**Keywords:** Influenza, Influenza hemagglutinin, Viral disease diagnosis, Infectious disease control, Immunologic techniques, Immunoassays, Monoclonal antibodies, Ficin digestion, Peptide mass fingerprinting, MALDI-TOF/TOF MS

## Abstract

**Background:**

The highly pathogenic avian influenza viruses of the H5 subtype, such as the H5N1 viral strains or the novel H5N8 and H5N2 reassortants, are of both veterinary and public health concern worldwide. To combat these viruses, monoclonal antibodies (mAbs) against H5 hemagglutinin (HA) play a significant role. These mAbs are effective diagnostic and therapeutic agents and powerful tools in vaccine development and basic scientific research. The aim of this study was to obtain diagnostically valuable mAbs with broad strain specificity against H5-subtype AIVs.

**Results:**

We applied the hybridoma method to produce anti-HA mAbs. The cloning and screening procedures resulted in the selection of 7 mouse hybridoma cell lines and their respective antibody clones. Preliminary immunoreactivity studies showed that these newly established mAbs, all of the IgG1 isotype, had high specificity and broad-range activities against the H5 HAs. However, these studies did not allow for a clear distinction among the selected antibodies and mAb-secreting hybridoma clones. To differentiate the analyzed mAbs and determine the exact number of hybridoma clones, peptide mapping of the Fc and Fab fragments was performed using a Matrix-Assisted Laser Desorption Ionization Time of Flight (MALDI-TOF/TOF) mass spectrometer. Detailed analyses of the acquired MS and MS/MS spectra confirmed that the Fc fragments constituted highly conserved species- and isotype-immunoglobulin components, whereas the Fab fragments exhibited considerable variation in the sequences that determine antibody specificity. This approach enabled unambiguous characterization of the selected mAbs according to their peptide composition. As a result, 6 different clones were distinguished.

**Conclusions:**

Our work provided a unique panel of anti-H5 HA mAbs, which meets the demand for novel, high-specificity analytical tools for use in serologic surveillance. Applications of these mAbs in areas other than diagnostics are also possible. Moreover, we demonstrated for the first time that peptide mapping of antibody fragments with mass spectrometry is an efficient method for the differentiation of antibody clones and relevant antibody-producing cell lines. The method may be successfully used to characterize mAbs at the protein level.

**Electronic supplementary material:**

The online version of this article (10.1186/s12985-017-0886-2) contains supplementary material, which is available to authorized users.

## Background

The highly pathogenic (HP) avian influenza viruses (AIVs) of the H5 subtype pose a serious epidemiological problem. This applies especially to H5N1 HPAIV, which was detected for the first time among farmed geese in China in 1996 and in humans a year later [1]. The spread of the H5N1 viruses to many regions of the world has been accompanied by frequent avian flu outbreaks in poultry that have resulted in mortality rates of up to 100% [2]. Moreover, as of January 2017, there have been a total of 856 laboratory-confirmed human cases of H5N1 influenza, 452 of which were fatal [3]. From 2009 onward, the emergence of the reassortant H5-subtype HPAIVs, such as H5N2, H5N5, H5N6 and H5N8, has been noted [2]. In addition, the novel H5N8 and H5N2 HPAIVs were observed to spread rapidly and globally soon after their identification in 2014 [4, 5]. Many populations of domestic birds have been substantially affected by these lethal viruses due to infection or mass culling [6, 7].

In view of the threat to animal and human health and life, the H5-subtype HPAIVs are under epidemiological surveillance. Various strategies have been developed for prevention and treatment of the infections caused by these viruses. The majority of the strategies have focused on the H5 hemagglutinin (HA), which determines the high pathogenicity of AIVs and is also the main target for neutralizing antibodies. In the efforts to combat H5-subtype HPAIVs, monoclonal antibodies (mAbs) against H5 HA play a significant role. When characterized by high specificity and affinity and/or neutralizing activities, they constitute effective diagnostic and therapeutic agents. Moreover, mAbs are powerful as tools for vaccine development as well as in basic research including studies of the antigenic architecture of the HA of influenza H5N1 viruses [8].

Most therapeutic and diagnostically valuable mAbs are immunoglobulins (Igs) of the G class (IgG). These are homodimeric glycoproteins of ~150 kDa. Each IgG molecule contains two identical heavy (H) chains and two identical (L) light chains with molecular weights of ~50 kDa and ~25 kDa, respectively [9]. The chains are connected by disulfide bonds to form a Y-shaped structure. Both the H and L chains of IgG antibodies consist of variable (V) and constant (C) domains referred to as VL and CL and VH, CH1, CH2 and CH3, respectively. The variable parts of the H and L chains, especially the complementarity-determining regions (CDRs), comprise the antigen-binding sites of the Ig molecules. They are responsible for the target epitope recognition, antigen-antibody reaction and the diversity of antibody specificity. Variations in mAbs may also result from post-translational modifications such as alternative disulfide pairings, deamidation, methionine oxidation or pyroglutamate formation. The heterogeneity of the glycosylation potently affects both pharmacokinetics and stability of various isoforms, which alter the clinical efficacy and safety of the therapeutic proteins [10].

Monoclonal antibodies can be generated using a hybridoma technique based on spleen/myeloma fusion. The concept was developed in the 1970s [11]. Another method involves the transformation of human B lymphocytes with the Epstein-Barr virus [12, 13]. Irrespective of the production method, the mAbs secreted by the immortalized cells need to be selected in terms of the desired function and/or specificity. In the case of mAbs against viral HAs, the hemagglutination inhibition (HI) and virus neutralization (VN) tests are used to ascertain the ability of established antibodies to confer protection against influenza. Identification of antibodies with the desired specificity is frequently accomplished by immunological techniques such as the enzyme-linked immunosorbent assay (ELISA). Alternative protocols that optimize the screening for antibody-producing cells using flow cytometry are also available [14, 15]. These techniques do not, however, provide complete information regarding the heterogeneity of the hybridoma clones.

In addition to the methods and techniques described above that have been applied to mAb production, mass spectrometry (MS) can be used. MALDI-TOF/TOF MS has been widely recognized in the fields of the development of therapeutic antibodies, determination of their structural features, glycan characterization and profiling [16–18]. The technique enables evaluation of the recombinant protein sequence and structure and provides information on amino acid modifications and sequence alterations. Although the resolution of the mass spectrometer is insufficient to differentiate between large, intact proteins, accurate measurements can be achieved at the peptide level [19].

To facilitate the structural analysis of antibodies, well known methods may be applied to selectively cleave the Ig molecules into fragments that have discrete characteristics and functions. If the variable regions of IgG antibodies are of primary interest, it is possible to generate their antigen-binding fragments, including the F(ab’)2, Fab’, Fab and Fv. For example, the monovalent fragment denoted Fab is composed of one constant and one variable domain of each of the H and the L chain, i.e., CH1, VH and CL, VL, respectively. The two variable domains, VH and VL, specifically bind the epitope of their respective antigen [20]. Preparation of the Fab fragments of IgG antibodies is usually accomplished by digestion of Igs with papain in the presence of a reducing agent [21]. In the case of mouse IgG1 antibodies, the enzyme of choice is ficin, applied with the optimized reductant concentration [22]. The Fab fragment does not contain any part of the crystallizable portion of the constant region of the Ig (Fc). Formed entirely from the H chain constant domains, the Fc fragment does not bind antigen and is responsible for the effector functions of antibodies.

In this paper, we describe newly generated, highly specific mAbs with a broad range of activities against the H5 HA of influenza viruses. As obtained, the mAbs were indistinguishable on the basis of the range of immunoreactivities determined by ELISA. We therefore employed MALDI-TOF/TOF MS as a structural tool for their differentiation. These mass measurements enabled assessment of the heterogeneity of peptide maps, obtained for the mAb-derived Fab and Fc fragments. Moreover, they provided clear distinctions among the analyzed clones and the antibody-secreting hybridoma cell lines. Thus, our results show for the first time that peptide mapping of antibody fragments with MS is an efficient alternative method for differentiation of antibody clones and the relevant antibody-producing cell lines.

## Methods

### Hemagglutinin antigens

This work was performed with the use of recombinant H5 HA proteins and inactivated AIVs of the H1-H16 subtypes, which are listed in Additional file 1: Tables S2 and S4, respectively. The ectodomain- or the HA1 subunit-based HA proteins (rHA, rHA1, respectively) were produced in a mammalian expression system (Immune Technology Corp., New York, NY, USA), except for one rHA protein, which was of baculovirus-expression system origin (Oxford Expression Technologies Ltd., Oxford, England, UK). Prior to use, the recombinant antigens were characterized by MS, ELISA for antigenicity and oligomerization and/or the hemagglutination test, performed as described in Additional file 1. The proteins were used to immunize mice, for plasma antibody titer determination, in preliminary or further specificity testing of hybridoma culture supernatants and/or reactivity studies of the finally selected, purified mAbs. The applications of each antigen are shown in Additional file 1: Table S2. Influenza viruses of the H5 (4 strains) and non-H5 subtypes (21 strains) were certified by Istituto Zooprofilattico Sperimentale delle Venezie (Legnaro, Padova, Italy) and originated from x-OvO Ltd. (Dunfermline, Scotland, UK). The viral strains of the H5-subtype were used to test the culture supernatants from the hybridoma clones, as shown in Additional file 1: Table S4. Both H5 and non-H5 AIVs were used in the studies of the finally selected, purified mAbs (Additional file 1: Table S4). The H5 HA antigens intended for antibody screening were chosen from those commercially available to obtain the panel of antigens with diverse amino acid sequences of the HA1 subunit. The antigenic diversity was determined by homology searches against the immunogen’s HA1 subunit using the BLAST program on NCBI. Complete information on the HA antigens, including their abbreviated names and relevant viral strains, is provided in Additional file 1.

### Hybridoma production and screening

Female, 6-week-old BALB/c mice (Mossakowski Medical Research Centre PAS, Warsaw, Poland) were first immunized subcutaneously with 10 μg of rHA - A/H5N1/Qinghai, emulsified with an equal volume of Complete Freund’s Adjuvant (Sigma-Aldrich, St. Louis, MO, USA) using the two-syringe method. Two weeks later, two subsequent 10 μg doses of the same immunogen were given by intraperitoneal injection in the absence of an adjuvant at 3-week interval. Thereafter, the mice received an additional intravenous dose of 10 μg of rHA protein in PBS and were euthanized 3 days later.

The mouse was chosen for fusion on the basis of the antibody titers against rHA - A/H5N1/Qinghai and rHA - A/H5N1/Poland using an ELISA of the plasma samples collected after the third immunization. The splenocytes were fused with mouse myeloma cells of SP2/0 line (ATCC, Rockville, MD, USA) in the presence of 50% PEG 1500 and 5% DMSO. The fused hybrid cells were cultured in RPMI-1640 medium containing FBS, L-glutamine, sodium pyruvate, and antibiotics (streptomycin, penicillin), with hypoxanthine, aminopterin and thymidine (HAT) as the selecting agents. The hybridomas were subcloned by the limited dilution method. To subclone, cells of each hybridoma were suspended in 5 mL of complete RPMI-1640 medium, counted and diluted to 10 or 5 cells *per* mL. The obtained suspension was transferred into 96-well plates (100 μL *per* well equivalent to 1 or 0.5 cell *per* well). The resulting hybridoma cell lines were grown in RPMI-1640 medium with the same supplements as the selection culture medium except for HAT. The reagents used for fusion and hybridoma culture were purchased from Sigma-Aldrich.

The hybridoma culture supernatants were screened for the presence of anti-H5 HA antibodies using ELISA. Both before and after subcloning, preliminary testing was performed using the rHA - A/H5N1/Qinghai and the rHA - A/H5N1/Poland as antigens. To select broadly reacting antibodies, the analyses were completed using the various H5 HA antigens as shown in Additional file 1: Tables S2 and S4. The recombinant H5 HA proteins from mammalian and baculovirus expression systems were coated on Ni-NTA strips (Qiagen, Hilden, Germany) and MediSorp plates (Nunc, Roskilde, Denmark), respectively, and the H5-subtype AIVs were coated on MaxiSorp plates (Nunc). The hybridoma culture supernatants were analyzed in the antigen-coated and also in the non-coated wells to control for non-specific binding. Commercial antibodies against H5 HA (mAb 8 in Additional file 1: Table S1) were used as a positive control. The blank control was the culture medium. Anti-mouse IgG (γ-chain specific) antibodies labeled with HRP (Sigma-Aldrich) were applied to detect the antigen-antibody complexes. At all stages of the procedure, the antibodies were isotyped using a commercial kit: “Mouse Monoclonal Antibody Isotyping Reagents” (ISO-2; Sigma-Aldrich).

The selected mAbs in the final set, a total of 7 clones, were purified from the hybridoma culture supernatants using “HiTrap Protein G HP” (GE Healthcare, Uppsala, Sweden) according to the manufacturer’s instructions. The purified mAbs were stored in PBS that contained sodium azide.

### Determination of monoclonal antibody immunoreactivity by ELISA

The reactivity of the finally selected and purified mAbs was studied using all of the HA antigens depicted in Additional file 1: Tables S2 and S4. To perform the tests, Ni-NTA strips (Qiagen) were coated with the rHA and rHA1 proteins (1 μg/mL in 1% BSA/PBS) and MaxiSorp plates (Nunc) with AIVs of H1-H16 subtypes (4000 hemagglutination units/mL in PBS), all by overnight incubation at 2–8 °C. Because they were supplied pre-blocked, the coated Ni-NTA strips were used without the blocking step. The coated MaxiSorp plates were blocked with 2% BSA/PBS. Thereafter, the mAbs diluted in 2% BSA/PBS were applied to the antigen-coated wells and also to the non-coated wells to control for non-specific binding. The assay was performed in the presence of other control samples. Commercial antibodies against H5 HA (mAb 8 in Additional file 1: Table S1) were used in antibody testing with H5 HA antigens and non-H5 subtype AIVs to serve as positive and negative controls, respectively. The blank control was the dilution buffer. In the assays for cross-reactivity, additional control was provided by testing the mAbs with non-H5 subtype AIVs in parallel with H5N3 and H5N9 viruses. The plates with tested and control samples were incubated overnight at 2–8 °C.

Detection of signals was accomplished using HRP-labeled, anti-mouse IgG (γ-chain specific) antibodies (Sigma-Aldrich). The secondary antibodies, diluted 1:1000 in 2% BSA/PBS, were incubated with the test plates for 1 h at 37 °C. The reactions were developed with TMB (Sigma-Aldrich) at room temperature for 30 min and subsequently stopped by adding a solution of H_2_SO_4_. The absorption was read at 450 nm using a μQuant microplate spectrophotometer (BioTek Instr. Inc., Winooski, VT, USA). For each antibody sample, the mean absorbance value for blank control samples was subtracted.

### Immobilized ficin digestion

The monoclonal antibodies were concentrated with the use of VivaSpin6 MWCO 10000 units (Sartorius Stedim Biotech GmbH, Goettingen, Germany) with simultaneous buffer exchange to PBS. The digestion of the mAbs with Immobilized Ficin (Pierce™ Mouse IgG_1_ Fab and F(ab’)_2_ Micro Preparation Kit; Thermo Scientific, Waltham, MA, USA) was performed according to the manufacturer’s protocol. For each mAb, a sample containing 250 μg of the protein was applied to the spin column tube containing the equilibrated enzyme. The digestion was conducted for 5 h. After separation by affinity chromatography, the two fractions, which contained the Fab and the mixture of Fc and undigested IgG, were concentrated using VivaSpin6 MWCO 5000 units (Sartorius Stedim Biotech GmbH) with simultaneous buffer exchange to PBS.

### Gel electrophoresis, MS measurements and data analysis

Non-reducing and non-boiled SDS-PAGE was performed using a 5% stacking gel, pH 6.8 and a 12.5% resolving gel, pH 8.8. The wells were loaded with 40 μL of the concentrated fractions mixed with an equal volume of sample buffer (62.5 mM Tris-HCl, pH 6.8; 25% glycerol; 1% Bromophenol Blue). Coomassie Brilliant Blue G-250 was used to visualize the proteins. The protein bands corresponding to the Fab, Fc and undigested IgG were excised from the gel, washed, reduced in the presence of 50 μL of 10 mM dithiothreitol at 60 °C for 45 min and alkylated with 50 μL of 50 mM iodoacetamide at room temperature in the dark for 60 min. The samples were further incubated with 30 μL of trypsin buffer (10 ng/mL; Promega, Madison, WI, USA, Cat. No. V5111) at 37 °C for 18 h. Prior to the MS analysis, the samples were evaporated to dryness and dissolved in 10 μL of 0.1% trifluoroacetic acid (TFA). All other general-use reagents were from Sigma-Aldrich.

The MALDI-TOF/TOF measurements were performed using reflector mode with a 4800 Plus instrument (Applied Biosystems, Waltham, MA, USA). α-Cyano-4-hydroxy-cinnamic acid dissolved in 50:50 water/acetonitrile (J.T. Baker, Deventer, The Netherlands) with 0.1% TFA (final concentration) was the matrix used. External calibration was achieved with a 4700 proteomics analyzer calibration mixture provided by Applied Biosystems. Each sample was spotted 5 times onto a 384 Opti-TOF MALDI plate and analyzed. Data Explorer Software, Version 4.9 (Applied Biosystems) was applied to process the acquired spectra. The peptide identification was accomplished using the Mascot search engine (Matrix Science Inc., Boston, MA, USA) against the Swiss-Prot and NCBInr sequence databases. A mass tolerance of 25 ppm and one missing cleavage site for the peptide mass fingerprinting (PMF) and a tolerance of 0.6 Da and one missing cleavage site for the MS/MS search were allowed. Carbamidomethyl was set as a fixed modification, glycosylation was considered as variable modification. The monoisotopic masses of the peptides unambiguously identified by Mascot are given as [M + H]^+^. For the peptides not assigned to a known protein, the masses of represent the average experimental monoisotopic mass and are given as [M + H]^+^. The data are presented as mass-to-charge ratio (m/z) values.

## Results

### Generation and selection of hybridoma clones

Monoclonal antibodies against the influenza virus HA were produced by a conventional hybridoma method that was developed in a mouse model [11]. For mouse immunization, a purified, recombinant, ectodomain-based HA protein (rHA) with the sequence derived from A/Bar-headed Goose/Qinghai/12/05(H5N1) strain of HPAIV was applied. The immunogen is referred to as rHA - A/H5N1/Qinghai (Additional file 1: Table S2). According to the manufacturer’s specifications, the majority of the protein exists as trimer/oligomer forms. Prior to immunization, the quality of the rHA protein was verified by studies on its antigenicity, oligomerization status and the ability to bind to sialic acid-containing receptors. Our studies proved that rHA - A/H5N1/Qinghai comprises conformational epitopes targeted by H5-subtype specific, HI and/or VN antibodies and forms functional oligomers (Additional file 1: Figures S3 and S6, Table S3). This led to the conclusion that the tested rHA protein displays native-HA characteristics and is therefore a suitable immunogen for mAb production with hybridoma technology.

According to the procedure, the splenocytes of the mouse with the highest anti-HA plasma antibody titer were collected. The spleen/myeloma fusion resulted in 440 hybridomas, which were screened for the production of IgG antibodies against H5 HA by ELISA. The preliminary hybridoma selection was performed using two recombinant, ectodomain-based HA proteins as the antigens for the antibody testing. The first of these proteins was the rHA - A/H5N1/Qinghai that was described above as the immunogen. The other, denoted rHA - A/H5N1/Poland (Additional file 1: Table S2), was produced in a baculovirus expression system with the HA sequence of the A/swan/Poland/305-135 V08/2006(H5N1) strain of HPAIV. The antigen was highly homologous with the immunogen (Table 1). Conformational integrity of its HA1 subunit was confirmed using ELISA and/or hemagglutination tests (Additional file 1: Figure S4, Table S3).

Further positive selection of the hybridomas was performed using ectodomain- or HA1 subunit-based recombinant H5 HA proteins (rHA, rHA1, respectively) from a mammalian expression system (Additional file 1: Table S2). Prior to use, the rHA and rHA1 proteins were examined for antigenicity and oligomerization using ELISA in procedures similar to those applied to the antigens used for the preliminary hybridoma screening. These studies enabled discrimination between the properly folded and misfolded H5 HA proteins, which are further described as conformational and non-conformational antigens, respectively. According to the results presented in Additional file 1, the vast majority of recombinant H5 HA antigens contain well preserved conformational epitopes of the viral HA (Figure S5). Moreover, all conformational rHA proteins were found to form oligomeric structures (Additional file 1: Figure S6). This mimics the trimeric HA in a viral envelope. To complete the positive selection, certified, inactivated, H5-subtype AIVs were used (Additional file 1: Table S4).

The H5 HA antigens used in the subsequent stages of hybridoma screening are described in Additional file 1: Tables S2 and S4. Before hybridoma subcloning, the tests were performed using the rHA and rHA1 proteins with the HA sequences from the H5N1 virus strains, A/Bar-headed Goose/Qinghai/12/05, A/chicken/India/NIV33487/2006, A/swan/Poland/305-135 V08/2006, A/Vietnam/1203/2004 and A/goose/Guiyang/337/2006, and the H5N2 virus strain A/American green-winged teal/California/HKWF609/2007. After subcloning, the selection was performed using the rHA and rHA1 proteins based on the H5 HA sequences of the H5N1 virus strains, A/Bar-headed Goose/Qinghai/12/05, A/chicken/India/NIV33487/2006, A/swan/Poland/305-135 V08/2006, A/Vietnam/1203/2004, A/Hong Kong/156/97, A/Hong Kong/483/97, A/goose/Guiyang/337/2006 and A/chicken/Vietnam/NCVD-016/08 and the H5N2 virus strain A/American green-winged teal/California/HKWF609/2007. In addition, the H5N1, H5N2, H5N3 and H5N9 influenza viruses were used for testing. The combination of the applied procedure made it possible to efficiently select antibodies against H5 HAs and subsequently to define their specificity range.

The preliminary screening resulted in the selection of 58 hybridomas that produced antibodies that recognized rHA - A/H5N1/Qinghai from the initial 440 hybridomas (Additional file 2: Table S5). The antibodies produced by most of these hybridomas (50/58) were also capable of binding to rHA - A/H5N1/Poland. Further examination with the panel of recombinant proteins led to the selection of 25 hybridomas that secreted antibodies reactive with all of the antigens used for specificity testing, from which 6 were selected for subcloning (Additional file 2: Tables S5 and S6). After the subcloning, a total of 64 hybridoma cell lines were obtained (Additional file 2: Table S7). All clones were shown to produce mAbs of the same broad-range specificity against the conformation of the H5 HA antigens. None of the tested antibodies bound to the non-conformational antigen. For the final studies, 7 clones, representing 6 groups of hybridoma cell lines, were chosen. Thus, the clones denoted G-1-31-22, G-2-14-10, G-5-32-5, G-7-24-17 and G-7-27-18 were derived from the subcloning of the hybridomas designated G-1-31, G-2-14, G-5-32, G-7-24 and G-7-27, respectively. The exception was that the clones denoted G-6-42-42 and G-6-42-71 originated from the subcloning of the same hybridoma (G-6-42). The antibodies produced by the selected hybridoma cell lines were designated on the basis of the names of the respective hybridoma clones. Details of the hybridoma screening are provided in Additional file 2.

### Immunoreactivity of selected monoclonal antibodies

The selected G-1-31-22, G-2-14-10, G-5-32-5, G-6-42-42, G-6-42-71, G-7-24-17 and G-7-27-18 mAbs in the hybridoma culture supernatants were isotyped and then affinity purified and examined for specificity against the H5-subtype influenza viruses. The studies were performed using ELISAs against well-characterized HA antigens with diverse properties. The panel of antigens comprised recombinant proteins of various lengths, all produced in mammalian cells except for one rHA protein that was produced using a baculovirus expression system (Additional file 1: Table S2). In particular, the rHA - A/H5N1/Qinghai, rHA - A/H5N1/India, rHA - A/H5N1/Vietnam, rHA - A/H5N1/Guiyang, rHA - A/H5N2/California, rHA - A/H5N1/Ck/Vietnam, rHA - A/H5N1/Poland, rHA1 - A/H5N1/Vietnam, rHA1 - A/H5N1/HK/156 and rHA1 - A/H5N1/HK/483 proteins were used for testing. The majority of these antigens (9/10), including 6 rHA and 3 rHA1 proteins displayed native-HA characteristics (Additional file 1: Figures S3-S5). The rHA1 proteins existed as monomers and the rHA ones were at least partially present as oligomers (Additional file 1: Figure S6, Table S3). The content of oligomeric forms differed among the rHA antigens. For the antibody testing, certified, inactivated AIVs of the H5 subtype, i.e., H5N1, H5N2, H5N3 and H5N9, were also used (Additional file 1: Table S4).

The conformational antigens used in the specificity studies of selected mAbs included the sequences of 12 strains of the H5-subtype influenza viruses (Additional file 1: Tables S2 and S4). Among these were the H5N3 (1 strain), H5N9 (1 strain), H5N2 (2 strains) viruses and primarily, the H5N1 viruses (8 strains), which could be classified into 5 clades. Using the BLAST algorithm, it was established that the HA1 subunit of these H5 HA antigens shared from 88% to 99% amino acid sequence identity with immunogen’s HA1 subunit (Table 1). To ascertain the cross-reactivity of the generated mAbs, 21 strains of AIVs, which represented the H1-H4 and H6-H16 subtypes were used for testing (Additional file 1: Table S4). Similar to the H5-subtype influenza viruses, the AIVs of H1-H4 and H6-H16 subtypes were certified. The characteristics of the obtained antibody clones are presented in Table 1.

**Table 1:**
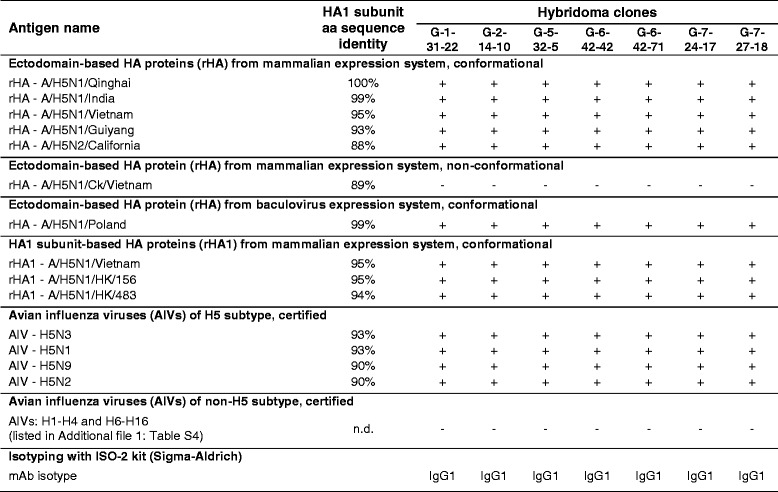
Results of preliminary immunoreactivity studies and isotyping of the finally selected monoclonal antibodies

The affinity-purified monoclonal antibodies (mAbs) were tested using ELISAs that targeted the recombinant proteins based on the ectodomain (rHA) or HA1 subunit (rHA1) of the H5 hemagglutinins (HAs) and the avian influenza viruses (AIVs) of H1-H16 subtypes as antigens. The HA antigens are listed in Additional file 1: Tables S2 and S4. The characteristics of the rHA and rHA1 proteins are presented in Additional file 1: Figures S1-S6 and Table S3. On this basis, the proteins were classified as conformational (properly folded) and non-conformational (misfolded) antigens. The amino acid sequence identities for the HA1 subunits of the H5 HAs were obtained using the BLAST program on NCBI by alignments against the 17–338-aa sequence of HA from the A/Bar-headed Goose/Qinghai/12/05(H5N1) HPAIV. The sequence homology with the non-H5 HAs was not determined (n.d.). The immunoreactivity studies and isotyping were performed in the presence of control samples as described in the Methods. The mean absorbance values for blank control samples were subtracted. Positivity and negativity in the tests with the specified antigens are indicated by plus and minus symbols, respectively. The underlying raw data are included in Additional file 3: Tables S8, S9 and S10

The finally selected mAbs, all of which were determined to be of the IgG1 isotype, recognized all of the conformational H5 HA antigens despite the substantial antigenic diversity among these target proteins. The target antigens included rHA (6/6) and rHA1 (3/3) proteins as well as H5-subtype AIVs (4/4). None of the clones bound to the misfolded rHA - A/H5N1/Ck/Vietnam protein. Thus, the results obtained for the purified mAbs were consistent with those obtained by testing the culture supernatants from the respective hybridoma cell lines (Additional file 2: Table S7). Furthermore, with the purified mAbs, we were able to show that none of selected clones bound to the AIVs of the H1-H4 and H6-H16 subtypes.

The results of our studies lead to the conclusion that these newly established mAbs are directed against epitopes in the properly folded HA1 subunit of the H5 HAs from highly divergent viral strains. Moreover, these mAbs did not cross-react with the influenza viruses of non-H5 subtypes. In summary, all of the obtained cloned antibodies were shown to have desirable properties but were poorly distinguished by the preliminary immunoreactivity tests.

### Differentiation of monoclonal antibodies by mass spectrometry

To characterize and group them, the selected mAbs were first subjected to immobilized ficin digestion and Protein A spin column separation. This procedure resulted in a flow-through fraction that contained the Fab fragments and an eluted fraction that contained the undigested IgG and the Fc fragments. Both protein fractions were separated using non-reducing and non-boiled SDS-PAGE (Additional file 4). Excision of the relevant protein bands, in-gel reduction, alkylation and trypsin digestion followed by MS analysis resulted in peptide maps of Fab and Fc fragments.

Detailed analyses of the maps permitted identification of peptides that were repeated in all of the antibody clones and peptides that occurred in only some of them. These two classes of peptides are further described as “common” and “discriminatory,” respectively. Furthermore, the “discriminatory” peptides that were found exclusively in one of analyzed antibody clones were further considered to be “unique” for this clone. To identify the peptides from the generated MS and MS/MS spectra, the Mascot search engine was used against the Swiss-Prot and NCBInr sequence databases. Thus, the term “identified” used in this context refers only to the peptides found in one or both of these databases. The peptide maps of the mAb-derived Fab and Fc fragments were characterized by the number and m/z values of the “common,” “discriminatory” and “unique” peptides as well as the number, m/z values and amino acid sequences of the peptides identified using Mascot. On this basis, the profiles of the tryptic peptide maps of individual antibody clones were defined. Attempts to differentiate the newly established mAbs and thereby the antibody-producing hybridoma cell lines were rationally focused on peptide maps of the Fab fragments that determine antibody specificity. It was hypothesized that differences in epitopic specificity of antibody clones would be reflected in divergence of their Fab fragment peptide maps.

#### Peptide maps of mAb-derived Fab fragments

Peptide mapping of Fab fragments from the G-1-31-22, G-2-14-10, G-5-32-5, G-6-42-42, G-6-42-71, G-7-24-17 and G-7-27-18 mAbs allowed the definition of a total of 23 peptides (Tables 2 and 3). Among these, 9 were “common” (Table 2) and 14 were “discriminatory” (Table 3) for the analyzed clones. The examined antibodies differed in the number of “discriminatory” peptides within their Fab fragments. Accordingly, 3 of these peptides were found in the G-2-14-10, G-6-42-42 and G-6-42-71 mAbs, 5 in the G-1-31-22 and G-5-32-5 mAbs, and 6 in the G-7-24-17 and G-7-27-18 mAbs. For a large majority of the tryptic peptides from Fab fragments, good quality MS and MS/MS spectra were obtained (Additional file 5). However, the Mascot search allowed for unambiguous identification of only 1 “common” and 3 “discriminatory” peptides. Most probably, this was due to the uniqueness of the analyzed amino acid sequences and the possible post-translational modifications of the Fab fragments, including glycosylation. The very limited number of peptides identified using Mascot is easy to understand given that newly established mAbs were being studied.

**Table 2:**
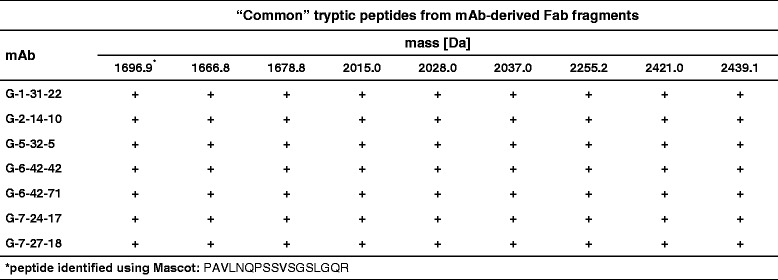
The characteristics of the “common” peptides in the tryptic maps of the Fab antibody fragments

The MALDI-TOF/TOF peptide maps were obtained from the tryptic digests of the Fab fragments excised from the non-reducing and non-boiled SDS-PAGE gel. The peptides were identified from the MS and MS/MS spectra using the Mascot search engine against the Swiss-Prot and NCBInr sequence databases. The identified peptide is marked with an asterisk symbol. By definition, the “common” peptides were present in the maps of all of the analyzed antibody clones, as indicated by plus symbols

**Table 3:**
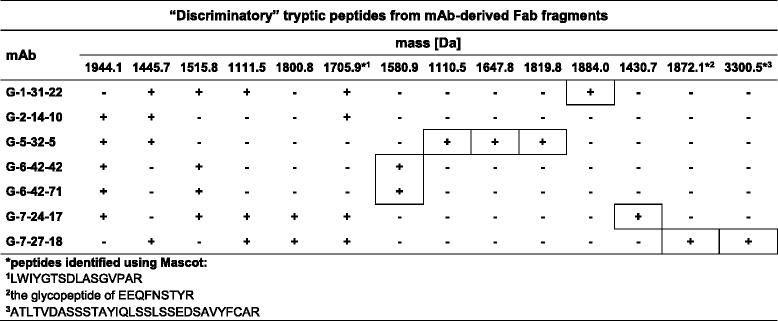
The characteristics of the “discriminatory” peptides in the tryptic maps of the Fab antibody fragments

The peptide mapping of the Fab fragments and identification of the amino acid sequences were performed as described in the legend to Table 2. The identified peptides are marked with asterisks followed by the reference numbers of respective amino acid sequences. The minus symbol indicates the absence of a given “discriminatory” peptide in the antibody map. The presence of a “discriminatory” peptide is denoted as a plus symbol. To distinguish the presence of a “unique” peptide, the plus symbol is framed

#### Profiles of “discriminatory” peptides in the Fab fragments

After the antibody clones were grouped, they were subjected to a further detailed comparative analysis based on the data presented in Table 3. Only the Fab fragment maps were examined. Thus, the same set of 3 “discriminatory” peptides was found in maps of the G-6-42-42 and G-6-42-71 antibodies. This indicates that G-6-42-42 and G-6-42-71 mAbs are the same clone. In the corresponding Fab fragments of these antibodies, a “unique” peptide at m/z 1580.9 was recognized. The simultaneous and exclusive presence of the peptides with m/z 1515.8 and 1944.1 additionally distinguished the G-6-42-42 and G-6-42-71 mAbs from the remaining 5 clones.

In the tryptic digest of the Fab fragment of the G-2-14-10 clone, 3 “discriminatory” peptides were obtained, including one peptide identified using Mascot (m/z 1705.9). For this mAb, no “unique” peptide was recognized. Analysis of the map generated for the G-5-32-5 clone indicated 5 “discriminatory” peptides, including 3 “unique” for the clone, which had m/z values of 1110.5, 1647.8 and 1819.8. The maps of both the G-2-14-10 and G-5-32-5 antibodies were characterized by the presence of 2 peptides with m/z 1445.7 and 1944.1. Subsequently, the investigation of the map for the G-1-31-22 clone demonstrated 5 “discriminatory” peptides, including 1 “unique” peptide with an m/z of 1884.0 and 1 that was identified using Mascot (m/z 1705.9). The G-1-31-22 mAb was characterized by the simultaneous presence of 3 peptides at m/z 1111.5, 1445.7 and 1515.8. The analysis of the data for the G-7-24-17 clone identified 6 “discriminatory” peptides, with 1 “unique” peptide at m/z 1430.7 and 1 that was identified using Mascot (m/z 1705.9). The G-7-24-17 mAb could be described by the presence of 4 signals at m/z 1111.5, 1515.8, 1800.8 and 1944.1. In the G-7-27-18 clone map, 6 “discriminatory” peptides were distinguished. The three at m/z 1705.9, 1872.1 and 3300.5 were identified using Mascot. The last two peptides were found to be “unique” for this clone. The G-7-27-18 mAb was differentiated by the simultaneous presence of 3 peptides at m/z 1111.5, 1445.7 and 1800.8.

#### Peptide maps of mAb-derived Fc fragments

The main mAb discrimination was completed based the on the profiles of the “discriminatory” peptides in the Fab fragment maps, as described above. To extract all possible data, a screening of Fc fragment-derived peptides was also performed. As a result, a total of 13 peptides were recognized in the maps of the G-1-31-22, G-2-14-10, G-5-32-5, G-6-42-42, G-6-42-71, G-7-24-17 and G-7-27-18 antibody clones (Table 4). Among these, 9 were “common” and 4 were “discriminatory” for the analyzed mAbs. According to data in Table 4, the G-6-42-42 and G-6-42-71 mAbs shared the same profile of “discriminatory” peptides within their Fc fragments. This confirms the indication previously shown in the profiling of the Fab-derived peptides (Table 3) that G-6-42-42 and G-6-42-71 mAbs represent a single antibody clone subsequently designated G-6-42-42,71. In two mAb groups, identical profiles of “discriminatory” peptides from the Fc fragments were recognized (Table 4). The first one comprises the previously mentioned G-6-42-42,71 antibodies and the G-7-24-17 mAb, and the second includes the G-2-14-10 and G-5-32-5 antibody clones. Compared to the second group, the G-7-27-18 mAb additionally contained a “unique” peptide. No “discriminatory” peptide was identified in the map of the G-1-31-22 clone Fc fragment. In the set of 13 peptides from the Fc fragments, the amino acid sequences of 9 were identified as fragments of IgG antibodies. These included 7 “common” and 2 “discriminatory” peptides.

**Table 4:**
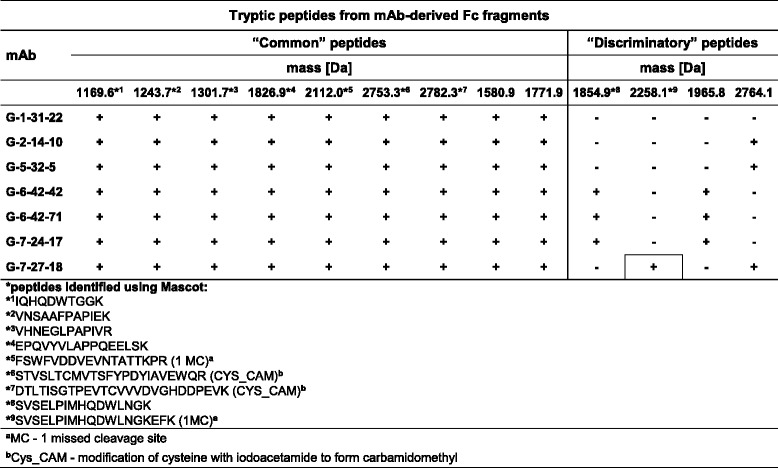
The characteristics of the peptides in the tryptic maps of the Fc antibody fragments

The MALDI-TOF/TOF peptide maps were obtained from the tryptic digests of the Fc fragments excised from non-reducing and non-boiled SDS-PAGE gel. The identification of the amino acid sequences was performed as described in the legend to Table 2. The identified “common” and “discriminatory” peptides are marked with asterisks followed by the reference numbers of the respective amino acid sequences. The plus and minus symbols indicate the presence and absence of a given peptide in the maps of the individual antibodies, respectively. To distinguish the presence of a “unique” peptide, the plus symbol is framed

#### The role of Fab and Fc fragment mapping in mAb differentiation

Consistent with the results of immunoreactivity and isotyping studies (Table 1), the peptide mapping of the mAb-derived fragments using MS showed that the analyzed Igs are not completely different. Individual “discriminatory” peptides were repeated in the Fab and Fc fragment maps of different antibodies along with the set of peptides “common” to all of them (Tables 2, 3 and 4). However, the presence or absence of the “unique” peptides within the Fab fragments and the pattern of the remaining “discriminatory” peptides enabled effective antibody differentiation (Table 3). Thus, 6 distinct clones were found among 7 newly established mAbs against the H5 HA. These 6 antibody clones probably recognize different epitopes in the antigen molecule. The peptide mapping of the Fc fragments from the anti-HA H5 IgG1 antibodies (Table 1) only grouped the antibody clones (Table 4) that were clearly distinguished by the profiling of the Fab-derived peptides (Table 3). On the other hand, this process allowed the definitive conclusion that the G-6-42-42 and G-6-42-71 mAbs, which shared the same Fab fragment maps, represent one antibody clone (Tables 2, 3 and 4).

## Discussion

Infection with HPAIVs of the H5 subtype leads to multi-organ disease and death in domestic birds [2, 4–7]. In addition, the H5N1 viral strains pose a persistent pandemic threat [3]. To prevent and treat H5N1 influenza virus infections and for surveillance of H5N1 and other H5-subtype AIVs, mAbs against H5 HA have been developed by many research groups (e.g., [23–28]; for review, see [8]). Our work responded to the demand for diagnostically valuable mAbs with broad strain specificity against AIVs of the H5 subtype. These antibodies were produced with hybridoma technology using recombinant, ectodomain-based H5 HA protein with native-HA characteristics to immunize mice.

The hybridomas generated by this process were screened for the production of IgG antibodies against the H5 HA using ELISA. The screening was performed against several forms of the HA antigen that had various properties. The use of conformational rHA1 proteins enabled the identification of antibodies that bound to the highly variable HA1 subunit, which determines the HA subtype. Distinguishing between conformation sensitive and non-sensitive antibodies was achieved using a misfolded rHA protein. In addition to the variations in the forms, the sequences of the HA antigens that were used originated from highly divergent H5-subtype influenza viruses. As a consequence, the H5 HA antigens demonstrated substantial antigenic diversity, which was confirmed by a homology search against the immunogen.

From our screening strategy, we obtained a total of 64 hybridoma cell lines. These cell lines secreted antibodies that were reactive with all of the H5 HA antigens that were used for the specificity testing except for the non-conformational antigen. A final set of 7 hybridoma clones was selected. Specifically, the G-1-31-22, G-2-14-10, G-5-32-5, G-6-42-42, G-6-42-71, G-7-24-17 and G-7-27-18 mAbs, all of IgG1 isotype, were further analyzed. In the preliminary immunoreactivity studies, we were able to show that the newly established mAbs specifically recognized epitopes in the properly folded HA1 subunit of H5 HAs from multiple strains of the H5-subtype influenza viruses (Table 1). Importantly, they did not cross-react with influenza viruses of H1-H4 and H6-H16 subtypes (Table 1). However, these studies did not allow for clear discrimination among the finally selected mAbs and the relevant hybridoma cell lines. For this reason, the exact number of the unique antibody and hybridoma clones could not be inferred.

The differentiation of antibody clones and relevant antibody-producing cell lines is of special importance for comprehensive assessment of their possible applications. When used in diagnostics or basic research, the set of mAbs that recognize different epitopes in the H5 HAs potentially extends the range of the target AIVs among the formerly and currently circulating viral strains. It can also facilitate the identification of the novel emerging H5-subtype AIVs. In addition, availability of different antibody clones enables the choice of the ones that will be best suited to specified method or technique. For example, two distinct mAbs can be successfully used as detection and capture antibodies in virus detection by a sandwich ELISA or immunochromatography.

Insight into the antibody heterogeneity could be provided by a comparison of the sequences encoding their variable regions, especially the CDRs [29]. Sequencing is routinely used to identify antibodies. However, it may be perceived as challenging if the presence of pseudogenes and mRNAs encoding non-functional antibody chains in hybridoma cells is considered [30]. Another method for differentiating mAbs is based on cross-inhibition experiments (e.g., [31]). Antibodies that do not compete for binding to the target antigen are considered to recognize distinct, non-overlapping epitopes. Competition between mAbs is interpreted as indicating that the tested antibody clones bind to the same or to closely related epitopes. Thus, cross-inhibition experiments may not give conclusive results. As the first mass spectra characterizing the generated antibody clones showed some differences between selected antibodies, we decided to expand them with peptide mapping of the Fab and Fc fragments. It was assumed that antibody examination at the protein level would allow to avoid some possible drawbacks related with their analyses at the genetic and functional levels.

Digestion with the immobilized ficin produced Fc and Fab fragments of the G-1-31-22, G-2-14-10, G-5-32-5, G-6-42-42, G-6-42-71, G-7-24-17 and G-7-27-18 mAbs. Subsequently, tryptic peptide maps of these fragments were generated. Based on the resulting MS and MS/MS spectra, Mascot searches against the Swiss-Prot and NCBInr sequence databases were performed. This enabled identification of some peptides derived from both Fc and Fab fragments, all of which belonged to the Ig class of proteins (Tables 2, 3, and 4).

Most of the peptides detected in the Fc fragments were “common” for the analyzed mAbs (69%; Table 4). The majority of the amino acid sequences of these fragments were identified within the protein databases. This is consistent with the widely accepted view that the Fc fragments are species- and isotype-conserved components of the Igs, which have no significance for their specificity [9]. In contrast, “discriminatory” peptides dominated the Fab fragment maps (61%; Table 3). Within these antibody fragments, very few sequences could be identified with the database searches: many fewer than for the conserved Fc fragments (17% vs. 69%; Tables 2, 3 and 4). These different proportions can be explained by the fact that the Fab fragments exhibit considerable variation in the specificity-determining sequences. For this reason, the protein databases are incomplete in this area.

A close inspection of the peptide maps of the Fab fragments revealed that the analyzed antibodies differed in the profiles of their “discriminatory” peptides (Table 3). Accordingly, 6 different clones were distinguished among the 7 selected mAbs. Presumably, these mAbs target distinct epitopes in the H5 HA molecule. For the G-6-42-42 and G-6-42-71 clones, identical peptide maps of the Fab and Fc fragments were obtained (Tables 2, 3 and 4). This indicates that these two mAbs are the same antibody clone. Interestingly, the G-6-42-42 and G-6-42-71 antibodies were the only clones among selected mAbs that originated from subcloning of the same hybridoma. Conclusions from the mass spectrometry approach are consistent with those from the advanced immunoreactivity studies (Additional file 6: Figures S10-S12).

### Addendum

On 9 June 2016, G-1-31-22, G-2-14-10, G-5-32-5, G-6-42-42, G-7-24-17, G-7-27-18 hybridoma cell lines were given the following Accession Numbers by the International Depositary Authority: DSM ACC3292, DSM ACC3293, DSM ACC3294, DSM ACC3295, DSM ACC3296 and DSM ACC3297, respectively. They are all held by the Leibniz Institute DSMZ-German Collection of Microorganisms and Cell Cultures (Braunschweig, Germany).

## Conclusions

A unique panel of 6 different anti-H5 HA antibody clones was generated and characterized. The newly established mAbs target epitopes in the properly folded HA1 subunit of HAs from multiple strains of the H5-subtype influenza viruses and do not cross-react with AIVs of H1-H4 and H6-H16 subtypes. Characterized by high specificity and broad-range activities against the H5 HAs, the described mAbs constitute valuable diagnostic and basic research tools. In the present study, the mass spectrometry approach has been developed as a method for antibody clone differentiation at the protein level. The method may be successfully used for characterization of mAbs that are poorly discriminated by immunological techniques as well as to obtain supplementary or confirmatory results. It also enables identification of unique antibody-producing cell lines.

## Additional files

Additional file 1: Antibodies and antigens used in this work. **Table S1.** Anti-H5 hemagglutinin antibodies. **Table S2.** Recombinant H5 hemagglutinin proteins. **Figure S1.** The MALDI-TOF/TOF mass spectrum of rHA - A/H5N1/Qinghai. **Figure S2.** The MALDI-TOF/TOF mass spectrum of rHA - A/H5N1/Poland. **Figure S3.** Antigenicity of rHA - A/H5N1/Qinghai. **Figure S4.** Antigenicity of rHA - A/H5N1/Poland. **Figure S5.** Antigenicity of recombinant hemagglutinin proteins. **Figure S6.** Oligomerization status of recombinant hemagglutinin proteins. **Table S3.** Hemagglutination activities of recombinant hemagglutinin proteins. **Table S4.** Avian influenza viruses. (PDF 752 kb)

Additional file 2: Results of hybridoma screening. **Table S5.** Reactivities of hybridoma culture supernatants with recombinant H5 hemagglutinin proteins. **Table S6.** ELISA absorbance values from testing of the selected hybridomas. **Table S7.** Reactivities of culture supernatants from the hybridoma cell lines with H5 hemagglutinin antigens. (PDF 349 kb)

Additional file 3: Raw data from the preliminary immunoreactivity studies. **Table S8.** The ELISA absorbance values for the selected mAbs tested against recombinant hemagglutinin antigens. **Table S9.** The ELISA absorbance values for the selected mAbs tested against AIVs of H1-H16 subtypes. **Table S10.** The ELISA absorbance values for the selected mAbs tested against AIVs of H5 subtype. (PDF 147 kb)

Additional file 4: SDS-PAGE data. (PDF 4249 kb)

Additional file 5: Exemplary MALDI-TOF mass spectra. (PDF 796 kb)

Additional file 6: Data from the advanced immunoreactivity studies. **Figure S7.** The ELISA titration curves of the mAbs against rHA - A/H5N1/Qinghai from a mammalian expression system. **Figure S8.** The ELISA titration curves of the mAbs against rHA - A/H5N1/Poland from a baculovirus expression system. **Figure S9.** The ELISA titration curves of the mAbs against rHA - A/H5N1/Poland from a bacterial expression system. **Table S11.** The concentration values interpolated from the mAb titration curves. **Figure S10.** Diversity of the mAb reactivities with recombinant H5 hemagglutinin proteins and H5N3 avian influenza virus. **Figure S11.** Relative reactivity of the mAbs with recombinant H5 hemagglutinin antigens. **Figure S12.** Relative reactivity of the mAbs with avian influenza viruses. (PDF 422 kb)

## Abbreviations

AIV: Avian influenza virus
CDR: Complementarity-determining region
ELISA: Enzyme-linked immunosorbent assay
Fab: Antigen-binding fragment
Fc: Crystallizable fragment
HA: Hemagglutinin
HI: Hemagglutination inhibition
HP: Highly pathogenic
Ig: Immunoglobulin
IgG: Immunoglobulin G class
mAb: Monoclonal antibody
MALDI-TOF/TOF MS: Matrix-Assisted Laser Desorption Ionization Time of Flight mass spectrometry
MS: Mass spectrometry
PMF: Peptide mass fingerprinting; m/z: mass-to-charge ratio
rHA: Ectodomain-based recombinant hemagglutinin protein
rHA1: HA1 subunit-based recombinant hemagglutinin protein
VN: Virus neutralization
