# Supplementary material for: Production of highly and broad-range specific monoclonal antibodies against hemagglutinin of H5-subtype avian influenza viruses and their differentiation by mass spectrometry
Source: Virol J. 2018 Jan 15;15:13. doi: 10.1186/s12985-017-0886-2 (PMC5769215; doi:10.1186/s12985-017-0886-2)
Supplement: Supplementary file 1 — Antibodies and antigens used in this work. Table S1. Anti-H5 hemagglutinin antibodies. Table S2. Recombinant H5 hemagglutinin proteins. Figure S1. The MALDI-TOF/TOF mass spectrum of rHA - A/H5N1/Qinghai. Figure S2. The MALDI-TOF/TOF mass spectrum of rHA - A/H5N1/Poland. Figure S3. Antigenicity of rHA - A/H5N1/Qinghai. Figure S4. Antigenicity of rHA - A/H5N1/Poland. Figure S5. Antigenicity of recombinant hemagglutinin proteins. Figure S6. Oligomerization status of recombinant hemagglutinin proteins. Table S3. Hemagglutination activities of recombinant hemagglutinin proteins. Table S4. Avian influenza viruses. (PDF 752 kb) [file 12985_2017_886_MOESM1_ESM.pdf]

Additional file 1: Antibodies and antigens used in this work

Anti-H5 hemagglutinin antibodies

In the examination of the hemagglutinin (HA) proteins intended for monoclonal antibody (mAb) production and/or characterization, a panel of commercially available antibodies (Abs) was used. A total of 8 antibody clones were purchased from USBiological, Salem, MA, USA (3 clones); Thermo Scientific, Waltham, MA, USA (1 clone); and Acris Antibodies, Herford, Germany (4 clones). Most of these mAbs (5/8) were specified as recognizing the H5 HAs in the hemagglutination inhibition test and as reactive with the H5N1, H5N2 and H5N9 influenza viruses and non-cross-reactive with HA of H1-H4 and H6-H15 subtypes. One of the 7 clones with HI activity was also characterized as being active in the virus neutralization test. According to the specifications, the polyclonal Abs (pAbs) from Immune Technology Corp. (New York, NY, USA) were directed against the HA1 or HA2 subunits of the HA from H5N1 influenza viruses.

The data provided by manufacturers on the mAbs and pAbs, denoted mAb 1 to mAb 8, pAb 1 and pAb2, are summarized in Table S1.

Table S1. Anti-H5 hemagglutinin antibodies.

Denotation	Source, Cat. No.	Immunogen	Isotype	Application
**Monoclonal antibodies (mAbs) against H5 hemagglutinin**				
**Recognizes H5 HA in the HI test. Reacts with H5N1, H5N2 and H5N9 strains. Does not cross-react with other HA types (H1, H2, H3, H4, H6, H7, H8, H9, H10, H11, H12, H13, H14 and H15).**				
**mAb 1**	USBiological, 17649-41B	Purified AIV type A (H5N1)	Ms IgG2a*	HI, iELISA, DB
**Recognizes the H5 HA protein of influenza A virus [strain A/Vietnam/1203/04(H5N1)]. Does not cross-react with influenza viruses of other HA subtypes. Reacts with H5N1 influenza virus representatives of different clades and subclades of the H5 subtype.**				
**mAb 2**	USBiological, 17649-42C	HA of the A/Vietnam/1203/04(H5N1) influenza virus	Ms IgG2a*	HI, VN, ELISA, IP, IHC, WB
**Recognizes H5 HA in HI test.**				
**mAb 3**	USBiological, 17649-42D	Purified AIV type A (H5N1)	Ms IgG2a*	HI, ELISA
**Detects influenza A H5 antigen in viral samples.**				
**mAb 4**	Thermo Scientific, MA1-81927	Purified AIV type A (H5N1)	Ms IgG2a*	ELISA
**React with H5 HA in HI test. React with H5N1, H5N2 and H5N9 strains. Do not cross-react with other HA types (H1, H2, H3, H4, H6, H7, H8, H9, H10, H11, H12, H13, H14 and H15).**				
**mAb 5**	Acris Antibodies, AM00942PU-N	Purified AIV type A (H5N1)	Ms IgG2a*	HI, iELISA, DB
**mAb 6**	Acris Antibodies, AM00944PU-N	Purified AIV type A (H5N1)	Ms IgG2a*	HI, iELISA, DB
**mAb 7**	Acris Antibodies, AM00945PU-N	Purified AIV type A (H5N1)	Ms IgG2a*	HI, iELISA, DB
**mAb 8**	Acris Antibodies, AM00941PU-N	Purified AIV type A (H5N1)	Ms IgG2a*	HI, iELISA, DB
**Polyclonal antibodies (pAbs) against H5 hemagglutinin**				
**Reacts with human and avian H5 (H5N1). Cross-reactivity to other HAs not tested.**				
**pAb 1**	Immune Technology Corp., IT-003-005G	HA (aa 1-345) (H5N1) (A/Bar-headed Goose/Qinghai/12/05(H5N1))	Rb IgG*	WB
**Reacts with HA and HA2 (H5N1). Cross-reactivity against other subtypes not tested.**				
**pAb 2**	Immune Technology Corp., IT-003-010	HA2 (H5N1) protein (aa 347-523) (A/VietNam/1203/2004(H5N1))	Rb IgG*	WB

Ms - Mouse; Rb – Rabbit; *purified; HI - hemagglutination inhibition test; iELISA - indirect ELISA (enzyme-linked immunosorbent assay); DB - dot blot; VN - virus neutralization test; IP - immunoprecipitation; IHC - immunohistochemistry; WB - Western blot.

Ability of mAb 7 and mAb 8 to inhibit hemagglutination was confirmed by us. The HI test was performed using chicken erythrocytes and H5N3 AIV (x-OvO Ltd.) as antigen.

Commercially available monoclonal and polyclonal antibodies (mAbs and pAbs, respectively) against the influenza virus H5 hemagglutinin (HA), denoted mAb 1 to mAb 8, pAb 1 and pAb 2, were used in the antigenicity studies of the H5 HA proteins.

Recombinant H5 hemagglutinin proteins

Recombinant H5 HA proteins were employed at various steps of the mAb production. For mouse immunization, the protein designated rHA - A/H5N1/Qinghai (Immune Technology Corp.) was used. The immunogen, which corresponds to the full-length ectodomain of HA, was expressed in mammalian cells with the cleavage-site deletion (ΔRRRKKR) and 6x His-tag at the C-terminus. The protein was then purified to approximately 95% purity. In the procedure for mAb production, other ectodomain-based proteins, denoted rHA, as well as the HA1 subunit-based proteins, denoted rHA1, from a mammalian expression system (Immune Technology Corp.) were used. In addition, one rHA protein, designated rHA - A/H5N1/Poland produced using a baculovirus expression system (Oxford Expression Technologies Ltd., Oxford, England, UK) was used.

The rHA and rHA1 proteins intended for the hybridoma screening were chosen from those commercially available to obtain a panel of antigens with diverse amino acid sequences, especially within the HA1 subunit. To achieve this aim, the BLAST program on NCBI was used to perform the sequence analysis against HA1 subunit of rHA - A/H5N1/Qinghai protein. Finally, the panel included the H5 HA antigens, characterized as having high to relatively weak homology with the immunogen. The sequences of the recombinant proteins originated from a total of 9 strains: 8 strains of H5N1 and 1 strain of H5N2 influenza viruses.

The H5 HA recombinant antigens were characterized by various methods: mass spectrometry and ELISAs for antigenicity and oligomerization as well as the hemagglutination test. The mass spectra of the rHA - A/H5N1/Qinghai and rHA - A/H5N1/Poland proteins were produced using a MALDI-TOF/TOF instrument (4800 Plus, Applied Biosystems, Waltham, MA, USA). The antigenicity studies of the rHA and rHA1 proteins were performed using the set of mAbs and pAbs shown in Table S1. These analyses were accomplished by coating MediSorp, MaxiSorp (Nunc, Roskilde, Denmark) and/or Ni-NTA (Qiagen, Hilden, Germany) ELISA plates with the target proteins to obtain the recognition profiles of the commercially available Abs for the rHA and rHA1 proteins. These are described in the paper entitled "A Novel Hemagglutinin Protein Produced in Bacteria Protects Chickens against H5N1 Highly Pathogenic Avian Influenza Viruses by Inducing H5 Subtype-specific Neutralizing Antibodies" by Sączyńska V et al. (PLoS One. 2017;12(2):e0172008. doi: 10.1371/journal.pone.0172008). For the present studies, the most important test was the one that discriminated between the properly folded and misfolded H5 HA antigens. The oligomerization status for all of the proteins was examined using a sandwich ELISA with non-labeled and HRP-labeled mAb 8 (Table S1) for plate coating and detection, respectively. The ability of the rHA - A/H5N1/Qinghai and rHA - A/H5N1/Poland proteins to evoke hemagglutination was tested using SPF chicken erythrocytes (National Veterinary Research Institute, Pulawy, Poland).

The data on the recombinant H5 HA proteins used for hybridoma generation and screening as well as the reactivity studies of the finally selected, purified mAbs are summarized in Table S2**.**

Table S2. Recombinant H5 hemagglutinin proteins.

**Antigen name** (test method / procedure stage)	**Relevant viral strain** Hemagglutinin sequence Genbank Accession No. (EpiFluDatabase Accession No.)	**Protein fragment** expression system (source)
**Ectodomain-based HA proteins (rHA)**		
rHA - A/H5N1/Qinghai (a, b, c, d / 1-3, 6)	A/Bar-headed Goose/Qinghai/12/05(H5N1), clade 2.2 ABE68927	17-530 aa, ΔRRRKKR, 6x His mammalian (ITC)
rHA - A/H5N1/India (b, c / 4-6)	A/chicken/India/NIV33487/2006(H5N1), clade 2.2 ABQ45850	17-530 aa, ΔRRRKKR, 6x His mammalian (ITC)
rHA - A/H5N1/Vietnam (b, c / 4-6)	A/Vietnam/1203/2004(H5N1), clade 1 AAW80717	18-530 aa, ΔRRRKKR, 6x His mammalian (ITC)
rHA - A/H5N1/Guiyang (b, c / 4-6)	A/goose/Guiyang/337/2006(H5N1), clade 4 ABJ96698	17-530 aa, ΔRRRKKR, 6x His mammalian (ITC)
rHA - A/H5N1/Ck/Vietnam (b, c / 5, 6)	A/chicken/Vietnam/NCVD-016/08(H5N1), clade 7 ACO07033	18-534 aa, 6x His mammalian (ITC)
rHA - A/H5N2/California (b, c / 4-6)	A/American green-winged teal/California/HKWF609/ 2007(H5N2) ACF47563	19-506 aa, 6xHis mammalian (ITC)
rHA - A/H5N1/Poland (a, b, c, d / 2, 3, 6)	A/swan/Poland/305-135V08/2006(H5N1), clade 2.2 (EPI156789)	17-530 aa, ΔRRRKKR, 6x His baculovirus (OET)
**HA1 subunit-based HA proteins (rHA1)**		
rHA1 - A/H5N1/Vietnam (b, c / 4-6)	A/Vietnam/1203/2004(H5N1), clade 1 AAW80717	1-345 aa, 6x His mammalian (ITC)
rHA1 - A/H5N1/HK/156 (b, c / 5, 6)	A/Hong Kong/156/97(H5N1), clade 0 AAC32088	18-346 aa, 6x His mammalian (ITC)
rHA1 - A/H5N1/HK/483 (b, c / 5, 6)	A/Hong Kong/483/97(H5N1), clade 0 AAC32099	17-346 aa, 6x His mammalian (ITC)

ITC - Immune Technology Corporation (USA); OET - Oxford Expression Technologies Ltd. (UK).

Based on the ectodomain (rHA) or the HA1 subunit (rHA1), the hemagglutinin (HA) proteins were characterized by (a) mass spectrometry, ELISAs for (b) antigenicity and (c) oligomerization and/or (d) the hemagglutination test. The individual proteins were used at the following stages of mAb production and characterization: (1) mouse immunization, (2) plasma antibody titer determination, (3) preliminary or (4, 5) additional specificity testing of the culture supernatants collected (3, 4) before and (3, 5) after hybridoma subcloning, and (6) reactivity studies of the finally selected, purified mAbs.

The characteristics of the rHA and rHA1 proteins are presented in Figures S1-S6 and Table S3.

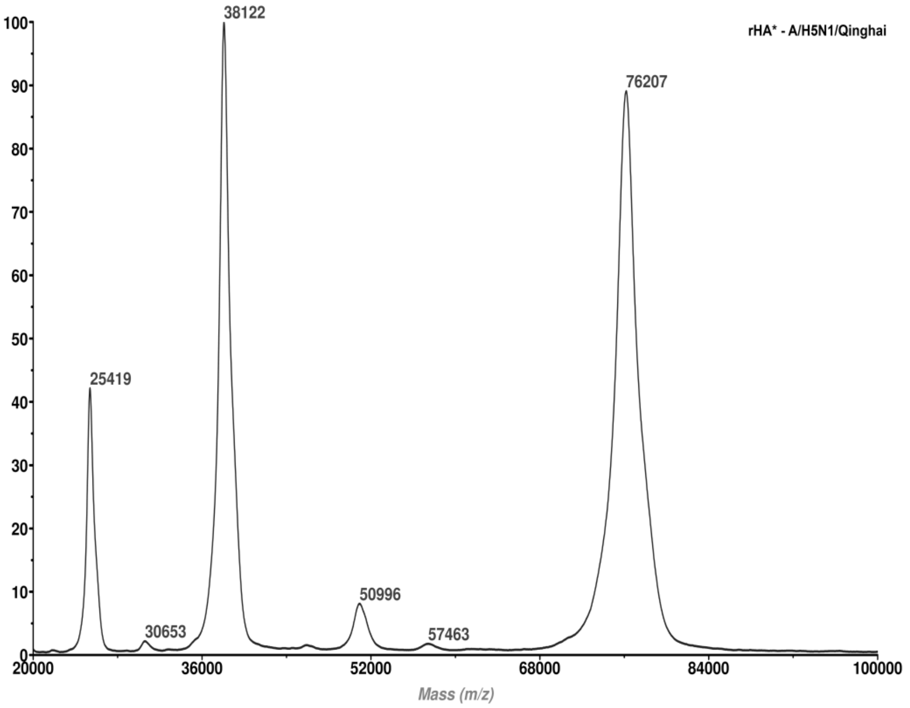

Figure S1. The MALDI-TOF/TOF mass spectrum of rHA - A/H5N1/Qinghai.

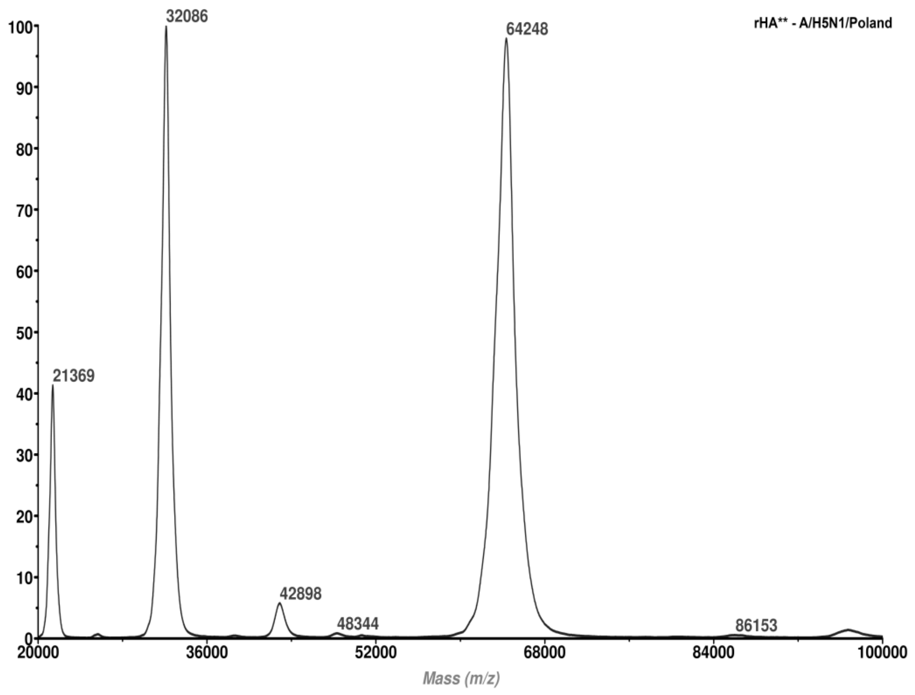

Figure S2. The MALDI-TOF/TOF mass spectrum of rHA - A/H5N1/Poland.

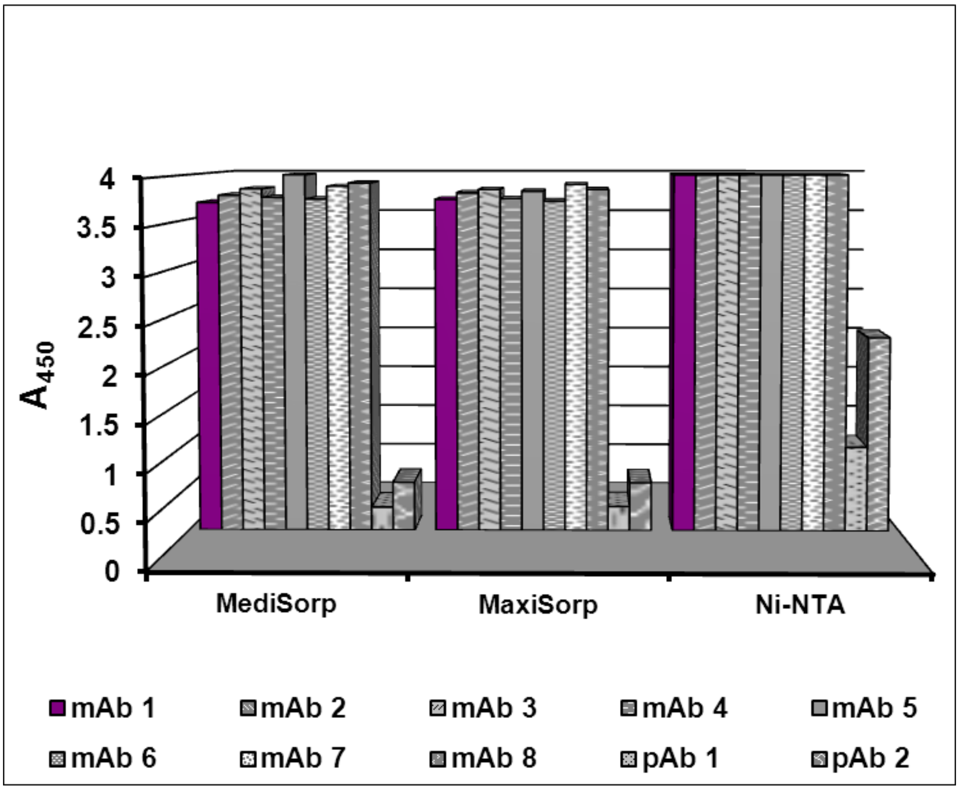

Figure S3. Antigenicity of rHA - A/H5N1/Qinghai.
The protein was coated at 5 µg/mL in PBS on MediSorp and MaxiSorp plates (Nunc) or at 1 µg/mL in 1% BSA/PBS on Ni-NTA strips (Qiagen). Antibodies (Table S1) were used at the concentration of 1 µg/mL in 2% BSA/PBS.

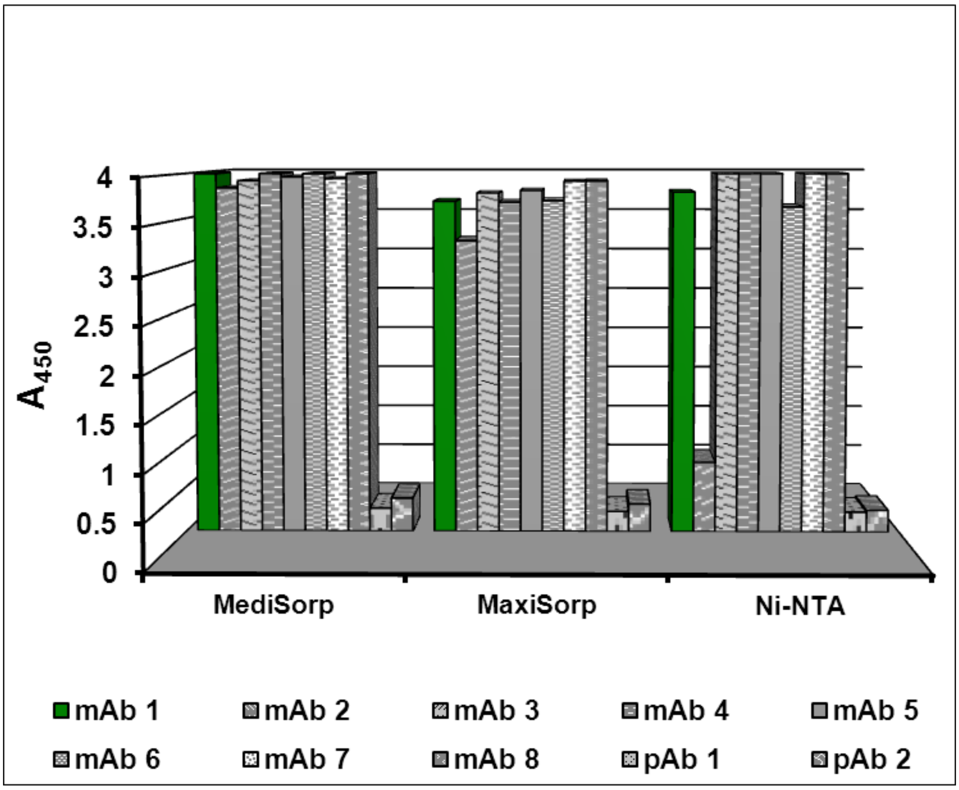

Figure S4. Antigenicity of rHA - A/H5N1/Poland.
The protein was coated at 5 µg/mL in PBS on MediSorp and MaxiSorp plates (Nunc) or at 1 µg/mL in 1% BSA/PBS on Ni-NTA strips (Qiagen). Antibodies (Table S1) were used at the concentration of 1 µg/mL in 2% BSA/PBS.

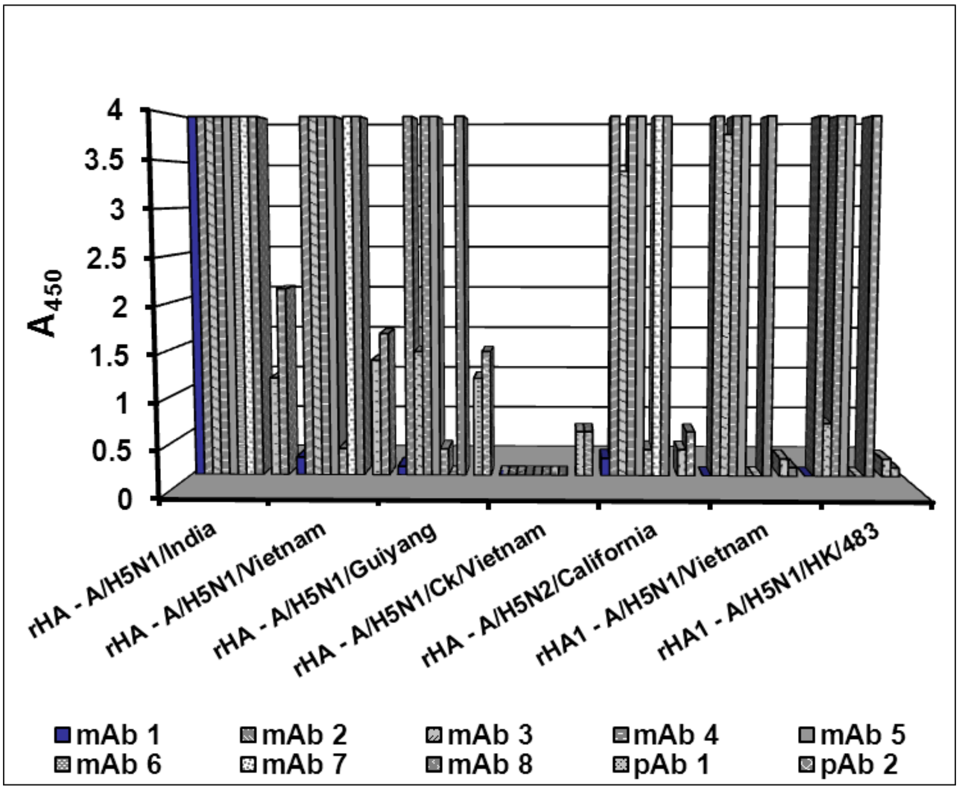

Figure S5. Antigenicity of recombinant hemagglutinin proteins.
The rHA and rHA1 proteins (Table S2) were coated on Ni-NTA strips (Qiagen) at 1 µg/mL in 1% BSA/PBS. Antibodies (Table S1) were used at the concentration of 1 µg/mL in 2% BSA/PBS.

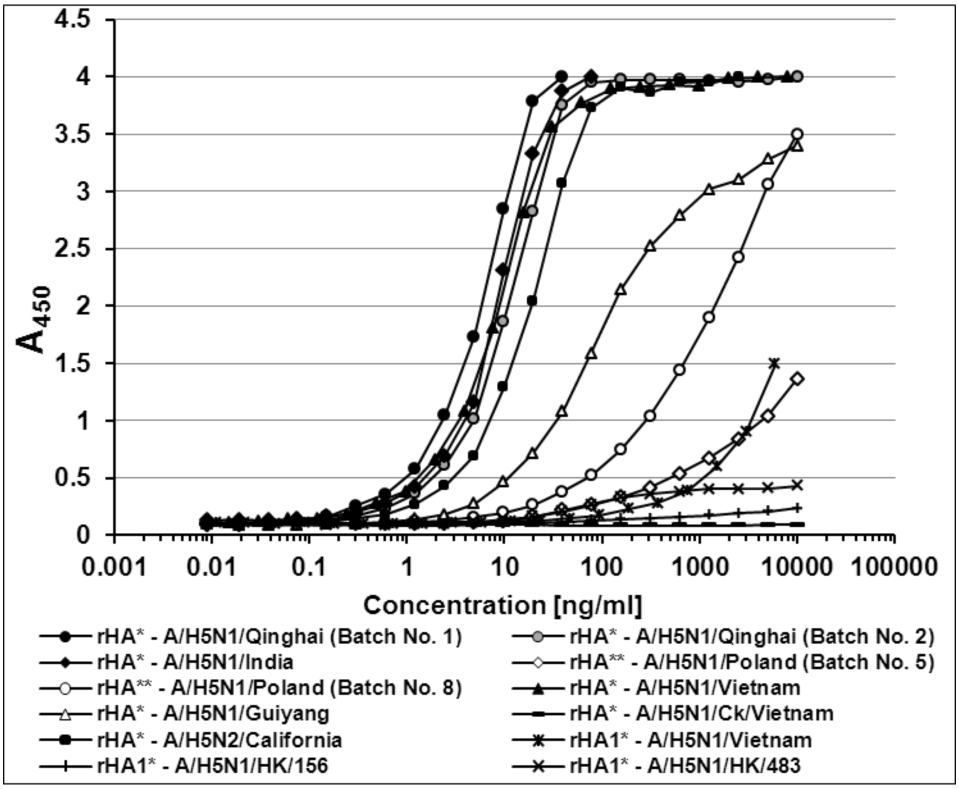

Figure S6. Oligomerization status of recombinant hemagglutinin proteins.
The rHA and rHA1 proteins from (*) mammalian or (**) baculovirus expression systems (Table S2) were examined by a sandwich ELISA using non-labeled and HRP-labeled antibodies of the same clone (mAb 8 in Table S1) as the capture and detection antibodies, respectively. Antibodies were coated at 1 µg/mL in PBS on MaxiSorp plates (Nunc). The proteins were analyzed after 2-fold serial dilution in 2% BSA/PBS. The labeled antibodies were used at a dilution of 1:500 in 2% BSA/PBS.

Table S3. Hemagglutination activities of recombinant hemagglutinin proteins.

Protein name	Hemagglutinin amount						
	10 µg	5 µg	2.5 µg	1 µg	0.5 µg	0.25 µg	0.1 µg
rHA - A/H5N1/Qinghai	+	+	+	+	+	+	+
rHA - A/H5N1/Poland	+	+	+	+/-	-	-	-

Hemagglutination activities of the rHA proteins (Table S2) were tested using chicken erythrocytes. (Data adapted from Sączyńska V et al. (2017). PLoS ONE 12(2): e0172008. doi:10.1371/journal.pone.0172008)

Avian influenza viruses

The panel of H5 HA antigens that were used in the antibody testing included the inactivated H5N1, H5N2, H5N3 and H5N9 avian influenza viruses (AIVs). This provided additional HA proteins in the native conformations and increased the amount of variability shown by the HA1 subunit as confirmed by a homology search (BLAST program on NCBI). Influenza viruses of the H5-subtype were used for the positive selection of hybridoma cell lines as well for the final immunoreactivity studies. The latter were performed using additional 21 strains of inactivated AIVs of non-H5 subtypes, i.e., H1-H4 and H6-H16. All of the viruses used were certified by the Istituto Zooprofilattico Sperimentale delle Venezie (Legnaro, Padova, Italy) and originated from x-OvO Ltd. (Dunfermline, Scotland, UK).

The data on the AIVs used for the positive and negative selection of mAbs are summarized in Table S4.

Table S4. Avian influenza viruses.

**Hemagglutinin**	**Avian influenza virus**		**Stage**
**Subtype**	**Subtype**	**Strain**	
**H1**	H1N1	A/duck/It/1447/05(H1N1)	**2**
**H2**	H2N3	A/duck/Germ/1215/73(H2N3)	**2**
**H3**	H3N8	A/pass/It/6000/V00(H3N8)	**2**
		A/psitt/It/2873/00(H3N8)	**2**
**H4**	H4N8	A/cockatoo/Eng/72(H4N8)	**2**
**H5**	H5N1	A/mallard/It/3401/05(H5N1), clade EA-nonGsGD	**1, 2**
	H5N2	A/turk/It/80(H5N2)	**1, 2**
	H5N3	A/duck/It/775/04(H5N3)	**1, 2**
	H5N9	A/ck/It/22A/98(H5N9)	**1, 2**
**H6**	H6N2	A/turkey/Canada/65 (H6N2)	**2**
**H7**	H7N1	A/ck/It/1067/V99(H7N1)	**2**
	H7N3	A/ty/It/9289/V02(H7N3)	**2**
	H7N4	A/mallard/It/4810-79/04(H7N4)	**2**
	H7N7	A/macaw/626/80(H7N7)	**2**
**H8**	H8N4	A/turk/Ont/6118/68(H8N4)	**2**
**H9**	H9N2	A/ty/Wis/66(H9N2)	**2**
	H9N7	A/turk/Scotland/1/70(H9N7)	**2**
**H10**	H10N1	A/ostrich/SA/01(H10N1)	**2**
**H11**	H11N6	A/duck/Eng/56(H11N6)	**2**
	H11N9	A/duck/Memphis/546/174(H11N9)	**2**
**H12**	H12N5	A/duck/Alberta/60/76(H12N5)	**2**
**H13**	H13N6	A/gull/Maryland/704/77(H13N6)	**2**
**H14**	H14N5	A/mallard/Gurjev/263/82(H14N5)	**2**
**H15**	H15N9	A/shearwater/2576/79(H15N9)	**2**
**H16**	H16N3	A/gull/Denmark/68110/02(H16N3)	**2**

All viruses were from x-OvO Ltd. (UK), certified by Istituto Zooprofilattico Sperimentale delle Venezie (Italy).

Influenza viruses were used in the reactivity testing of (1) the culture supernatants from hybridoma clones and of (2) the finally selected, purified mAbs.
