# Supplementary material for: Production of highly and broad-range specific monoclonal antibodies against hemagglutinin of H5-subtype avian influenza viruses and their differentiation by mass spectrometry
Source: Virol J. 2018 Jan 15;15:13. doi: 10.1186/s12985-017-0886-2 (PMC5769215; doi:10.1186/s12985-017-0886-2)
Supplement: Supplementary file 2 — Results of hybridoma screening. Table S5. Reactivities of hybridoma culture supernatants with recombinant H5 hemagglutinin proteins. Table S6. ELISA absorbance values from testing of the selected hybridomas. Table S7. Reactivities of culture supernatants from the hybridoma cell lines with H5 hemagglutinin antigens. (PDF 349 kb) [file 12985_2017_886_MOESM2_ESM.pdf]

Additional file 2: Results of hybridoma screening

The culture supernatants from hybridomas and hybridoma cell lines were screened for the presence of anti-H5 HA antibodies using ELISAs that targeted the recombinant proteins based on the ectodomain (rHA) or HA1 subunit (rHA1) of the H5 hemagglutinins (HAs) and/or the H5-subtype avian influenza viruses (AIVs) as antigens. Details on HA antigens used for testing are provided in Additional file 1. Due to a very large dataset that was generated during hybridoma screening and selection procedure, the results were compiled in Tables S5 and S7 showing positivity and negativity in the tests performed before and after hybridoma subcloning. ELISA absorbance values from testing of the 6 hybridomas selected for subcloning are presented in Table S6.

The hybridoma screening enabled identification of the 25 hybridomas that secreted antibodies reactive with all of the antigens used for specificity testing (Table S5).

Table S5. Reactivities of hybridoma culture supernatants with recombinant H5 hemagglutinin proteins.

**Antigen name**	**Sequence identity**		**58 hybridomas**									
	**aa 1-567**	**aa 17-338**	**25**	**3**	**3**	**9**	**4**	**7**	**3**	**2**	**1**	**1**
**Ectodomain-based HA proteins (rHA) from mammalian expression system, conformational**												
rHA - A/H5N1/Qinghai	**100**	**100**	+	+	+	+	+	+	+	+	+	+
rHA - A/H5N1/India	99	99	+	+	+	+	+	+	+	+	+	+
rHA - A/H5N1/Vietnam	97	95	+	+	+	+	+	+	+	+	+	+
rHA - A/H5N1/Guiyang	94	93	+	+	-	+	+	+	+	-	-	-
rHA - A/H5N2/California	89	88	+	-	+	+	+	-	-	+	-	-
**Ectodomain-based HA protein (rHA) from baculovirus expression system, conformational**												
rHA - A/H5N1/Poland	99	99	+	+	+	+	-	+	-	+	+	-
**HA1 subunit-based HA proteins (rHA1) from mammalian expression system, conformational**												
rHA1 - A/H5N1/Vietnam	-	95	+	+	+	-	-	-	-	-	-	-
**Selection**			↓ **6 hybridomas**									

Culture supernatants were analyzed on Ni-NTA strips (Qiagen) coated with the rHA and rHA1 proteins from a mammalian expression system (1 µg/mL in 1% BSA/PBS) and on MediSorp plates (Nunc) coated with rHA - A/H5N1/Poland from a baculovirus expression system (5 µg/mL in PBS). To control for non-specific binding, the supernatants were also analyzed in the non-coated wells. Commercial antibodies against H5 HA (mAb 8 in Additional file 1: Table S1) were used as a positive control. The blank control was the culture medium. The antigen-antibody complexes were detected as described in the Methods. The mean absorbance values for blank control samples were subtracted. Positivity and negativity in the tests with the specified antigens are indicated by plus and minus symbols, respectively.

Hybridomas selected for subcloning produced antibodies with high activities against both the rHA and rHA1 proteins (Table S6). The latter reactivity is of special importance as the HA1 subunit determines subtype of influenza virus hemagglutinin.

Table S6. ELISA absorbance values from testing of the selected hybridomas.

**Antigen name**	**Sequence identity**		**6 hybridomas selected for subcloning**					
	**aa 1-567**	**aa 17-338**	**G-1-31**	**G-2-14**	**G-5-32**	**G-6-42**	**G-7-24**	**G-7-27**
**Ectodomain-based HA proteins (rHA) from mammalian expression system, conformational**								
rHA - A/H5N1/Qinghai	**100**	**100**	> 4.0	> 4.0	> 4.0	> 4.0	> 4.0	> 4.0
rHA - A/H5N1/India	99	99	> 4.0	> 4.0	> 4.0	> 4.0	> 4.0	> 4.0
rHA - A/H5N1/Vietnam	97	95	> 4.0	> 4.0	> 4.0	> 4.0	> 4.0	> 4.0
rHA - A/H5N1/Guiyang	94	93	> 4.0	> 4.0	> 4.0	> 4.0	> 4.0	> 4.0
rHA - A/H5N2/California	89	88	> 4.0	> 4.0	> 4.0	> 4.0	> 4.0	3.784
**Ectodomain-based HA protein (rHA) from baculovirus expression system, conformational**								
rHA - A/H5N1/Poland	99	99	3.143	2.594	3.220	3.343	3.104	3.335
**HA1 subunit-based HA proteins (rHA1) from mammalian expression system, conformational**								
rHA1 - A/H5N1/Vietnam	-	95	1.532	1.244	1.861	3.638	1.670	1.975

Culture supernatants were analyzed as described in the legend to Table S5. For each antibody sample, the mean absorbance value for blank control samples was subtracted. Thus obtained ELISA absorbance values are presented.

Hybridoma cell lines secreted antibodies with desirable activities against properly folded rHA and rHA1 proteins and H5-subtype AIVs (Table S7). Some differences between immunoreactivity profiles of antibodies produced by various-origin hybridoma cell lines were observed. Thus, clones originated from each of the 6 hybridomas were selected for the final studies.

Table S7. Reactivities of culture supernatants from the hybridoma cell lines with H5 hemagglutinin antigens.

Antigen name	Sequence identity		6 hybridomas					
			G-1-31	G-2-14	G-5-32	G-6-42	G-7-24	G-7-27
	aa 1-567	aa 17-338	64 hybridoma cell lines					
			22	1	10	6	14	11
**Ectodomain-based HA proteins (rHA) from mammalian expression system, conformational**								
rHA - A/H5N1/Qinghai	100	100	+	+	+	+	+	+
rHA - A/H5N1/India	99	99	+	+	+	+	+	+
rHA - A/H5N1/Vietnam	97	95	+	+	+	+	+	+
rHA - A/H5N1/Guiyang	94	93	+	+	+	+	+	+
rHA - A/H5N2/California	89	88	+	+	+	+	+	+
**Ectodomain-based HA protein (rHA) from mammalian expression system, non-conformational**								
rHA - A/H5N1/Ck/Vietnam	91	89	-	-	-	-	-	-
**Ectodomain-based HA protein (rHA) from baculovirus expression system, conformational**								
rHA - A/H5N1/Poland	99	99	+	+	+	+	+	+
**HA1 subunit-based HA proteins (rHA1) from mammalian expression system, conformational**								
rHA1 - A/H5N1/Vietnam	-	95	+	+	+	+	+	+
rHA1 - A/H5N1/HK/156	-	95	+	+	+	+	+	+
rHA1 - A/H5N1/HK/483	-	94	+	+	+	+	+	+
**Avian influenza viruses (AIVs) of H5 subtype, certified**								
IV - H5N3	93	93	+	+	+	+	+	+
IV - H5N1	93	93	+	+	+	+	+	+
IV - H5N9	91	90	+	+	+	+	+	+
IV - H5N2	91	90	+	+	+	+	+	+
**Isotyping with ISO-2 kit (Sigma-Aldrich)**								
**mAb isotype**			IgG1	IgG1	IgG1	IgG1	IgG1	IgG1
**Selection**			↓	↓	↓	↓	↓	↓
			1 clone	1 clone	1 clone	2 clones	1 clone	1 clone
			**G-1-31-22**	**G-2-14-10**	**G-5-32-5**	**G-6-42-42**	**G-7-24-17**	**G-7-27-18**
						**G-6-42-71**		

Culture supernatants were analyzed using indicated H5 HA antigens. The rHA and rHA1 proteins from a mammalian expression system were coated on Ni-NTA strips (Qiagen) at 1 μg/mL and 5 μg/mL in 1% BSA/PBS, respectively. The rHA - A/H5N1/Poland from a baculovirus expression system was coated on MediSorp plates (Nunc) at 5 μg/mL in PBS. The H5-subtype AIVs were coated on MaxiSorp plates (Nunc) at 4000 hemagglutination units/mL in PBS. To control for non-specific binding, the supernatants were also analyzed in the non-coated wells. Commercial antibodies against H5 HA (mAb 8 in Additional file 1: Table S1) were used as a positive control. The blank control was the culture medium. The antigen-antibody complexes were detected as described in the Methods. The mean absorbance values for blank control samples were subtracted. Positivity and negativity in the tests with the specified antigens are indicated by plus and minus symbols, respectively. The antibodies were isotyped using a commercial kit: “Mouse Monoclonal Antibody Isotyping Reagents” (ISO-2; Sigma-Aldrich).
