# Supplementary material for: Production of highly and broad-range specific monoclonal antibodies against hemagglutinin of H5-subtype avian influenza viruses and their differentiation by mass spectrometry
Source: Virol J. 2018 Jan 15;15:13. doi: 10.1186/s12985-017-0886-2 (PMC5769215; doi:10.1186/s12985-017-0886-2)
Supplement: Supplementary file 3 — Raw data from the preliminary immunoreactivity studies. Table S8. The ELISA absorbance values for the selected mAbs tested against recombinant hemagglutinin antigens. Table S9. The ELISA absorbance values for the selected mAbs tested against AIVs of H1-H16 subtypes. Table S10. The ELISA absorbance values for the selected mAbs tested against AIVs of H5 subtype. (PDF 147 kb) [file 12985_2017_886_MOESM3_ESM.pdf]

Additional file 3: Raw data from the preliminary immunoreactivity studies

The newly established monoclonal antibodies (mAbs) were tested using ELISAs that targeted the recombinant proteins based on the ectodomain (rHA) or HA1 subunit (rHA1) of the H5 hemagglutinins and the avian influenza viruses (AIVs) of H1-H16 subtypes as antigens (Additional file 1). The same concentrations of individual antibody clones were used for testing. The tests were performed as described in the Methods. The resulting characteristics of the obtained antibody clones are described in the manuscript and summarized in Table 1. The underlying raw data are included in Tables S8, S9 and S10.

Table S8. The ELISA absorbance values for the selected mAbs tested against recombinant hemagglutinin antigens.

Antigen name	Hybridoma clones						
	G-1-31-22	G-2-14-10	G-5-32-5	G-6-42-42	G-6-42-71	G-7-24-17	G-7-27-18
**Ectodomain-based HA proteins (rHA) from mammalian expression system, conformational**							
rHA - A/H5N1/Qinghai	2.440	3.749	3.786	> 4	> 4	2.281	> 4
rHA - A/H5N1/India	2.865	4.000	4.000	> 4	> 4	2.579	> 4
rHA - A/H5N1/Vietnam	2.789	3.922	4.000	> 4	> 4	2.474	> 4
rHA - A/H5N1/Guiyang	3.003	> 4	> 4	> 4	> 4	2.746	> 4
rHA - A/H5N2/California	1.726	3.183	3.566	3.666	3.125	1.514	4.000
**Ectodomain-based HA protein (rHA) from mammalian expression system, non-conformational**							
rHA - A/H5N1/Ck/Vietnam	0.000	0.004	0.004	0.005	0.006	0.002	0.009
**Ectodomain-based HA protein (rHA) from baculovirus expression system, conformational**							
rHA - A/H5N1/Poland	1.437	2.692	3.221	3.339	2.663	1.613	3.475
**HA1 subunit-based HA proteins (rHA1) from mammalian expression system, conformational**							
rHA1 - A/H5N1/Vietnam	2.024	3.427	3.724	> 4	3.557	1.940	> 4
rHA1 - A/H5N1/HK/156	2.334	3.588	3.613	3.784	3.268	2.110	4.000
rHA1 - A/H5N1/HK/483	1.588	2.979	3.262	3.508	2.755	1.420	3.742

The affinity-purified mAbs were analyzed at 0.05 μg/mL in 2% BSA/PBS on Ni-NTA strips (Qiagen) coated with the recombinant H5 hemagglutinin proteins (1 μg/mL in 1% BSA/PBS). To control for non-specific binding, the mAbs were also analyzed in the non-coated wells. Commercial antibodies against H5 HA (mAb 8 in Additional file 1: Table S1) were used as a positive control. The blank control was the dilution buffer. The mean absorbance values for blank control samples were subtracted.

Table S9. The ELISA absorbance values for the selected mAbs tested against AIVs of H1-H16 subtypes.

Hemagglutinin	Avian influenza virus		Hybridoma clones						
Subtype	Subtype	Strain	G-1-31-22	G-2-14-10	G-5-32-5	G-6-42-42	G-6-42-71	G-7-24-17	G-7-27-18
**H1**	**H1N1**	A/duck/It/1447/05(H1N1)	0.033	0.014	0.000	0.000	0.000	0.019	0.053
**H2**	**H2N3**	A/duck/Germ/1215/73(H2N3)	0.042	0.084	0.005	0.000	0.003	0.032	0.112
**H3**	**H3N8**	A/pass/It/6000/V00(H3N8)	0.016	0.051	0.000	0.000	0.000	0.010	0.074
		A/psitt/It/2873/00(H3N8)	0.024	0.018	0.000	0.000	0.000	0.017	0.052
**H4**	**H4N8**	A/cockatoo/Eng/72(H4N8)	0.022	0.029	0.002	0.000	0.000	0.011	0.053
**H5**	**H5N1**	A/mallard/It/3401/05(H5N1)	0.932	0.826	0.457	1.145	1.126	0.548	1.037
	**H5N2**	A/turk/It/80(H5N2)	0.368	0.360	0.229	0.419	0.404	0.204	0.395
	**H5N3**	A/duck/It/775/04(H5N3)	1.312	1.474	1.218	1.524	1.717	1.070	1.650
	**H5N9**	A/ck/It/22A/98(H5N9)	0.440	0.453	0.326	0.160	0.158	0.356	0.483
**H6**	**H6N2**	A/turkey/Canada/65 (H6N2)	0.038	0.039	0.002	0.000	0.002	0.022	0.074
**H7**	**H7N1**	A/ck/It/1067/V99(H7N1)	0.072	0.024	0.000	0.000	0.000	0.027	0.062
	**H7N3**	A/ty/It/9289/V02(H7N3)	0.043	0.012	0.002	0.001	0.004	0.023	0.055
	**H7N4**	A/mallard/It/4810-79/04(H7N4)	0.059	0.030	0.005	0.001	0.003	0.032	0.087
	**H7N7**	A/macaw/626/80(H7N7)	0.035	0.038	0.003	0.000	0.000	0.026	0.078
**H8**	**H8N4**	A/turk/Ont/6118/68(H8N4)	0.034	0.036	0.001	0.000	0.001	0.030	0.063
**H9**	**H9N2**	A/ty/Wis/66(H9N2)	0.020	0.030	0.000	0.000	0.000	0.010	0.048
	**H9N7**	A/turk/Scotland/1/70(H9N7)	0.010	0.023	0.000	0.000	0.000	0.006	0.031
**H10**	**H10N1**	A/ostrich/SA/01(H10N1)	0.016	0.003	0.000	0.000	0.003	0.014	0.028
**H11**	**H11N6**	A/duck/Eng/56(H11N6)	0.017	0.018	0.000	0.000	0.000	0.010	0.036
	**H11N9**	A/duck/Memphis/546/174(H11N9)	0.014	0.022	0.001	0.000	0.001	0.013	0.033
**H12**	**H12N5**	A/duck/Alberta/60/76(H12N5)	0.025	0.040	0.004	0.000	0.003	0.021	0.055
**H13**	**H13N6**	A/gull/Maryland/704/77(H13N6)	0.015	0.028	0.000	0.000	0.000	0.009	0.051
**H14**	**H14N5**	A/mallard/Gurjev/263/82(H14N5)	0.015	0.031	0.003	0.000	0.000	0.023	0.029
**H15**	**H15N9**	A/shearwater/2576/79(H15N9)	0.026	0.037	0.006	0.000	0.000	0.021	0.041
**H16**	**H16N3**	A/gull/Denmark/68110/02(H16N3)	0.053	0.059	0.007	0.000	0.000	0.028	0.095

The affinity-purified mAbs were analyzed at 20 µg/mL in 2% BSA/PBS on MaxiSorp plates (Nunc) coated with the avian influenza viruses (4000 hemagglutination units/mL in PBS). To control for non-specific binding, the mAbs were also analyzed in the non-coated wells. Testing the mAbs with non-H5 subtype AIVs was performed using in parallel the H5N3 and H5N9 viruses to provide additional controls. Commercial antibodies against H5 HA (mAb 8 in Additional file 1: Table S1) were used to serve as a positive control in the assays with H5-subtype AIVs and as a negative control in the assays with AIVs of the H1-H4 and H6-H16 subtypes. The blank control was the dilution buffer. The mean absorbance values for blank control samples were subtracted. The tests were performed in volumes of 50 µL *per* well.

Table S10. The ELISA absorbance values for the selected mAbs tested against AIVs of the H5 subtype.

Hemagglutinin	Avian influenza virus		Hybridoma clones						
Subtype	Subtype	strain	G-1-31-22	G-2-14-10	G-5-32-5	G-6-42-42	G-6-42-71	G-7-24-17	G-7-27-18
**H5**	H5N1	A/mallard/It/3401/05(H5N1)	1.166	1.162	0.623	1.604	1.624	0.782	1.573
	H5N2	A/turk/It/80(H5N2)	0.516	0.511	0.435	0.897	0.884	0.350	0.619
	H5N3	A/duck/It/775/04(H5N3)	1.460	1.913	1.381	2.966	3.036	1.438	2.345
	H5N9	A/ck/It/22A/98(H5N9)	0.549	0.651	0.489	0.315	0.291	0.560	0.723

The affinity-purified mAbs were analyzed at 20 μg/mL in 2% BSA/PBS on MaxiSorp plates (Nunc) coated with the avian influenza viruses (4000 hemagglutination units/mL in PBS). To control for non-specific binding, the mAbs were also analyzed in the non-coated wells. Commercial antibodies against H5 HA (mAb 8 in Additional file 1: Table S1) were used as a positive control. The blank control was the dilution buffer. The mean absorbance values for blank control samples were subtracted. The tests were performed in volumes of 100 μL *per* well.
