# Supplementary material for: Production of highly and broad-range specific monoclonal antibodies against hemagglutinin of H5-subtype avian influenza viruses and their differentiation by mass spectrometry
Source: Virol J. 2018 Jan 15;15:13. doi: 10.1186/s12985-017-0886-2 (PMC5769215; doi:10.1186/s12985-017-0886-2)
Supplement: Supplementary file 4 — SDS-PAGE data. (PDF 4249 kb) [file 12985_2017_886_MOESM4_ESM.pdf]

Additional file 4: SDS-PAGE data

To characterize and group them, the selected mAbs were first subjected to immobilized ficin digestion and Protein A spin column separation. This procedure resulted in a flow-through fraction that contained the Fab fragments and an eluted fraction that contained the undigested IgG and the Fc fragments. Both protein fractions were separated using non-reducing and non-boiled SDS-PAGE: a 5% stacking gel, pH 6.8 and a 12.5% resolving gel, pH 8.8 were exploited. The wells were loaded with 40 μL of the concentrated fractions mixed with an equal volume of sample buffer (62.5 mM Tris-HCl, pH 6.8; 25% glycerol; 1% Bromophenol Blue). Coomassie Brilliant Blue G-250 was used to visualize the proteins. Excision of the relevant protein bands, in-gel reduction, alkylation and trypsin digestion followed by MS analysis resulted in peptide maps of Fab and Fc fragments.

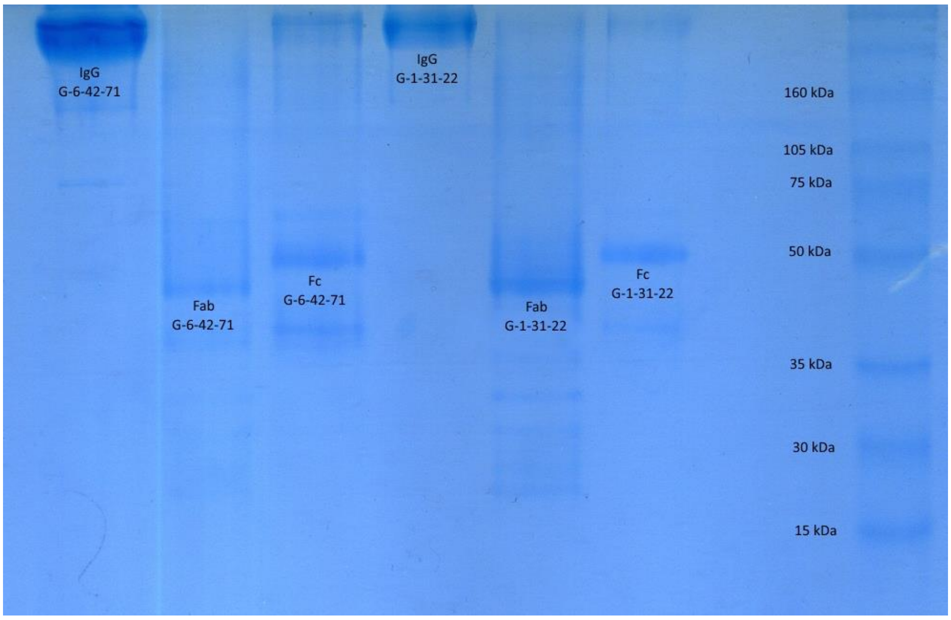

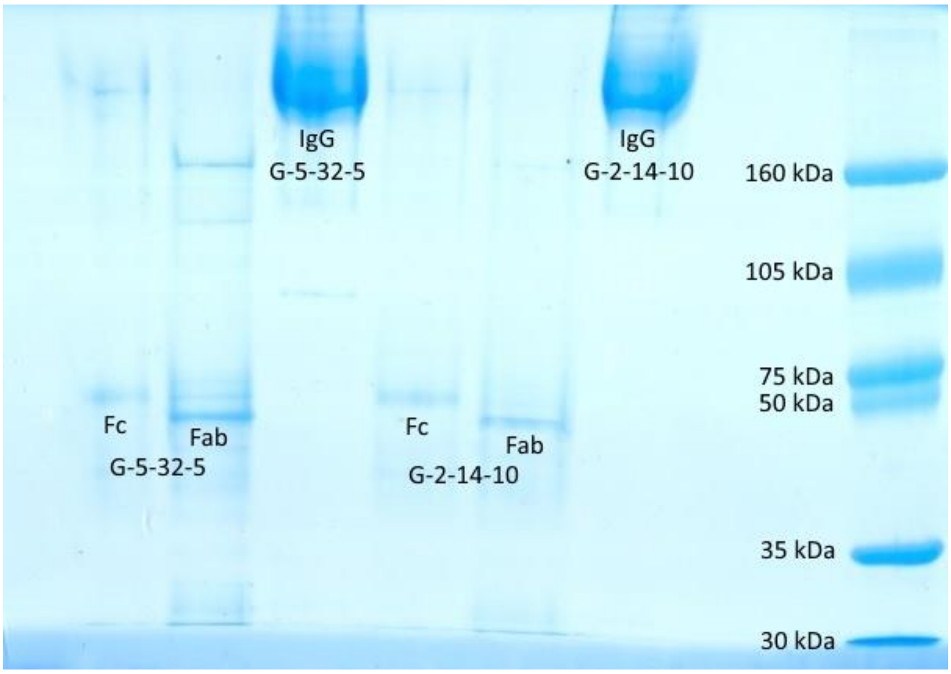
IgG
G-5-32-5
IgG
G-2-14-10
160 kDa
105 kDa
75 kDa
50 kDa
Fc
Fab
G-5-32-5
Fc
Fab
G-2-14-10
35 kDa
30 kDa

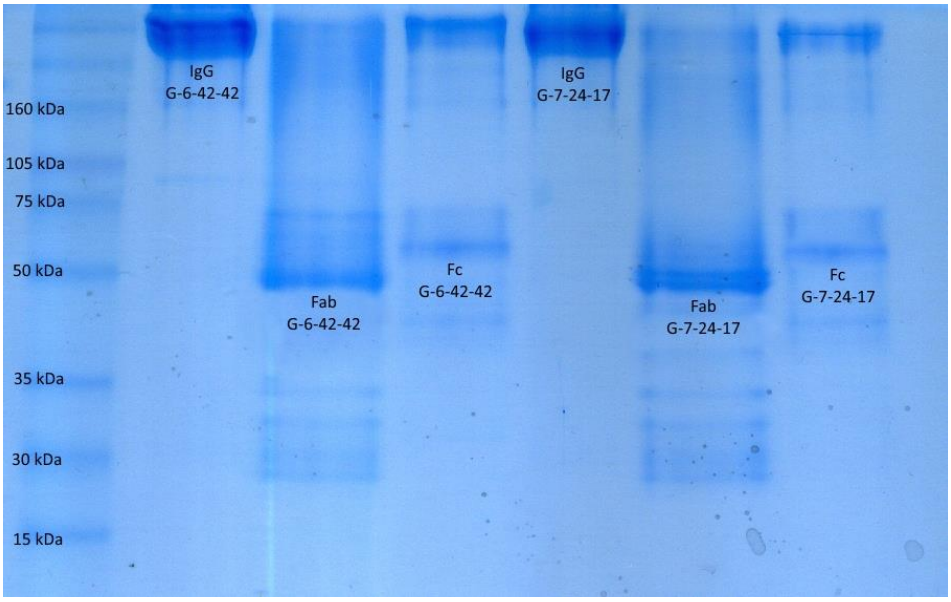
IgG
G-6-42-42
IgG
G-7-24-17
160 kDa
105 kDa
75 kDa
50 kDa
Fc
G-6-42-42
Fab
G-6-42-42
Fab
G-7-24-17
Fc
G-7-24-17
35 kDa
30 kDa
15 kDa

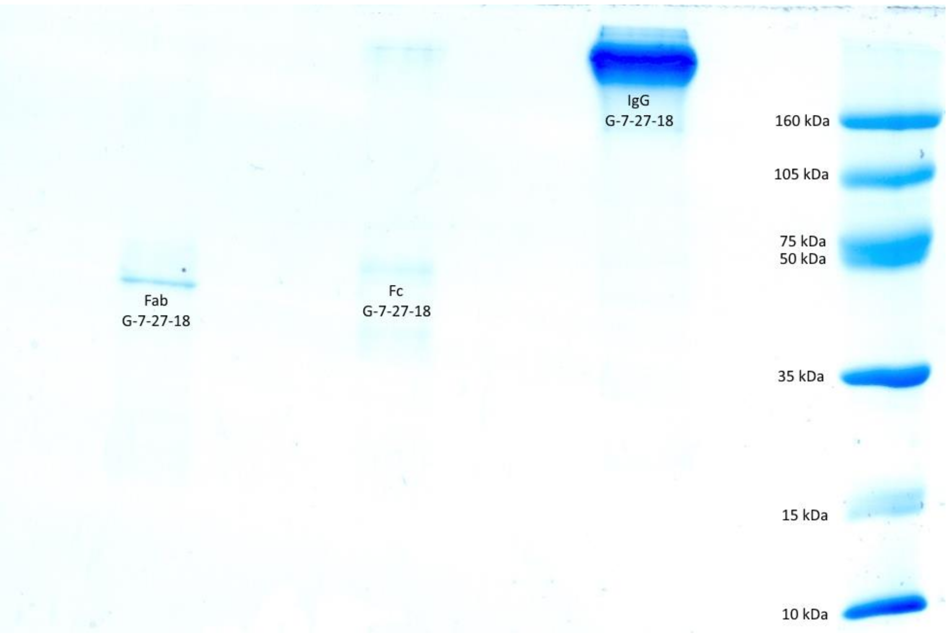
IgG
G-7-27-18
160 kDa
105 kDa
75 kDa
50 kDa
Fab
G-7-27-18
Fc
G-7-27-18
35 kDa
15 kDa
10 kDa
