# Supplementary material for: Production of highly and broad-range specific monoclonal antibodies against hemagglutinin of H5-subtype avian influenza viruses and their differentiation by mass spectrometry
Source: Virol J. 2018 Jan 15;15:13. doi: 10.1186/s12985-017-0886-2 (PMC5769215; doi:10.1186/s12985-017-0886-2)
Supplement: Supplementary file 5 — Exemplary MALDI-TOF mass spectra. (PDF 796 kb) [file 12985_2017_886_MOESM5_ESM.pdf]

Additional file 5: Exemplary MALDI-TOF mass spectra

Representative peptide maps obtained from the tryptic digests of the Fab fragments from the G-1-31-22, G-2-14-10, G-5-32-5, G-6-42-42, G-6-42-71, G-7-24-17 and G-7-27-18 mAbs are presented below. The corresponding protein bands were excised from the non-reducing and non-boiled SDS-PAGE gel, reduced and alkylated prior to cleavage with trypsin. The presented MALDI-TOF spectra were acquired using reflector mode with a 4800 Plus instrument (Applied Biosystems, Waltham, MA, USA). α-Cyano-4-hydroxy-cinnamic acid dissolved in 50:50 water/acetonitrile with 0.1% TFA (final concentration) was the matrix used.

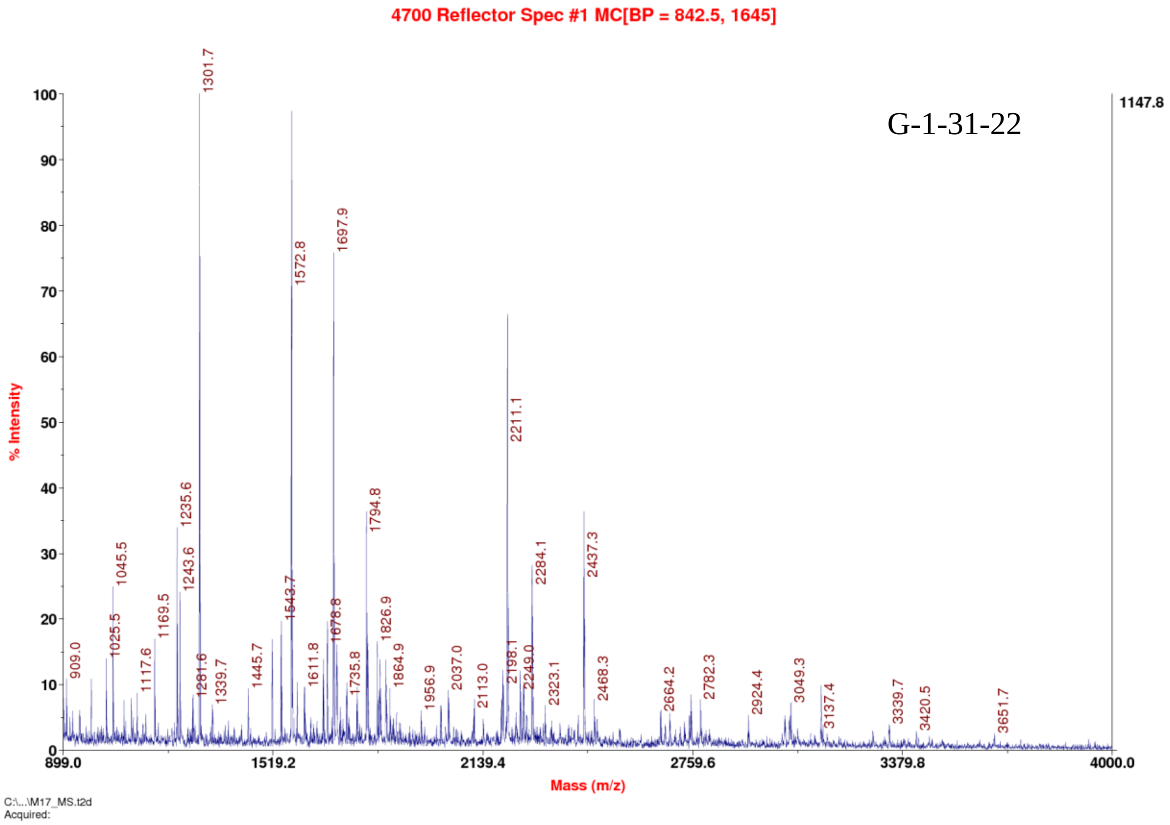

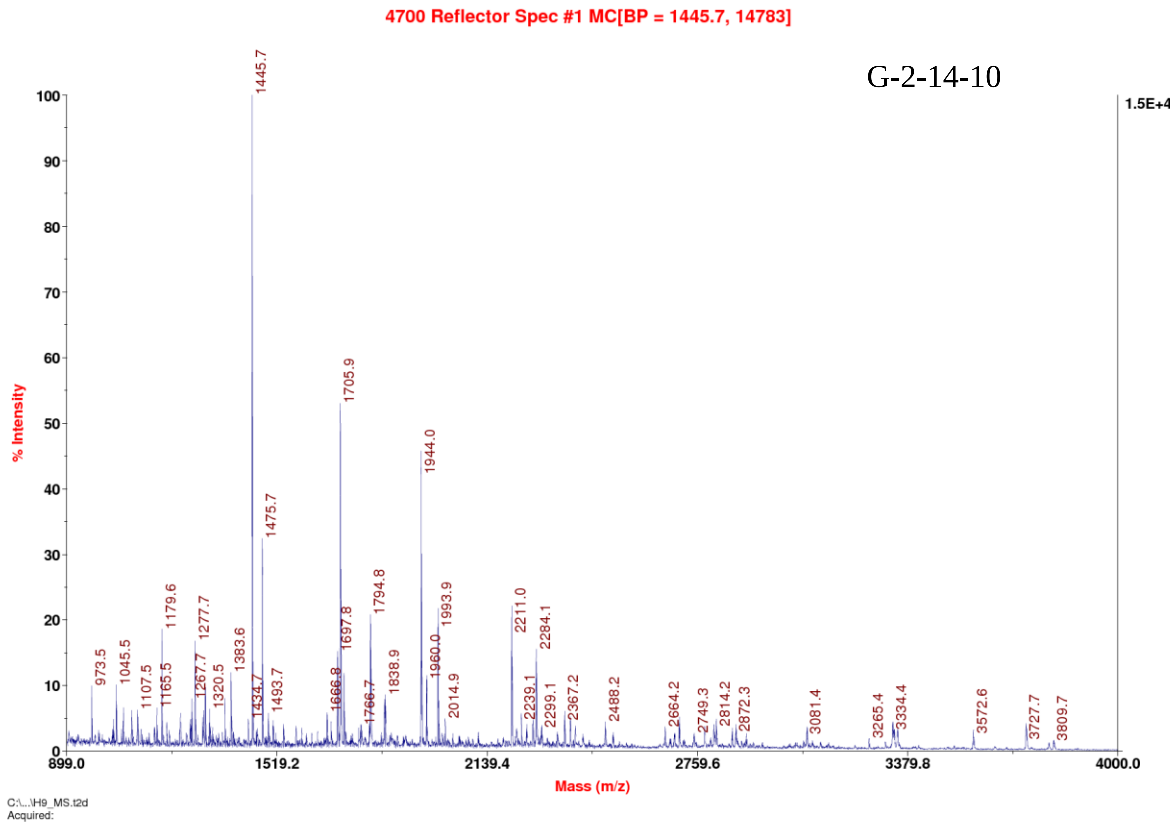
4700 Reflector Spec #1 MC[BP = 1445.7, 14783]
G-2-14-10
% Intensity
Mass (m/z)
1.5E+4
899.0
1519.2
2139.4
2759.6
3379.8
4000.0
1445.7
1705.9
1944.0
1475.7
1794.8
1993.9
2211.0
2284.1
1179.6
1277.7
1383.6
973.5
1045.5
1107.5
1165.5
1267.7
1320.5
1434.7
1493.7
1666.8
1697.8
1766.7
1838.9
1960.0
2014.9
2239.1
2299.1
2367.2
2488.2
2664.2
2749.3
2814.2
2872.3
3081.4
3265.4
3334.4
3572.6
3727.7
3809.7
C:\...\H9_MS.t2d
Acquired:

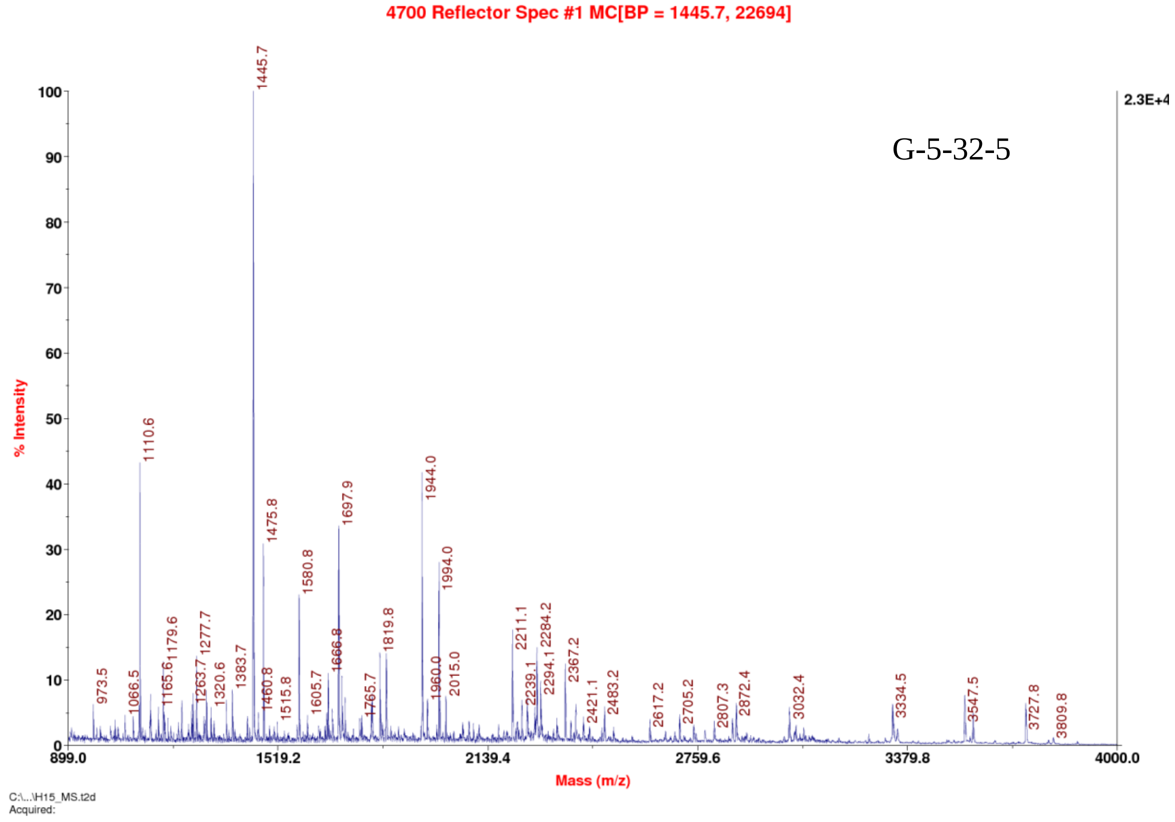
4700 Reflector Spec #1 MC[BP = 1445.7, 22694]
G-5-32-5
% Intensity
Mass (m/z)
2.3E+4
899.0
1519.2
2139.4
2759.6
3379.8
4000.0
1445.7
1110.6
1944.0
1475.8
1697.9
1994.0
1580.8
1819.8
2211.1
2284.2
1179.6
1277.7
973.5
1066.5
1165.6
1263.7
1320.6
1383.7
1460.8
1515.8
1605.7
1666.8
1765.7
1960.0
2015.0
2239.1
2294.1
2367.2
2421.1
2483.2
2617.2
2705.2
2807.3
2872.4
3032.4
3334.5
3547.5
3727.8
3809.8
C:\...\H15_MS.t2d
Acquired:

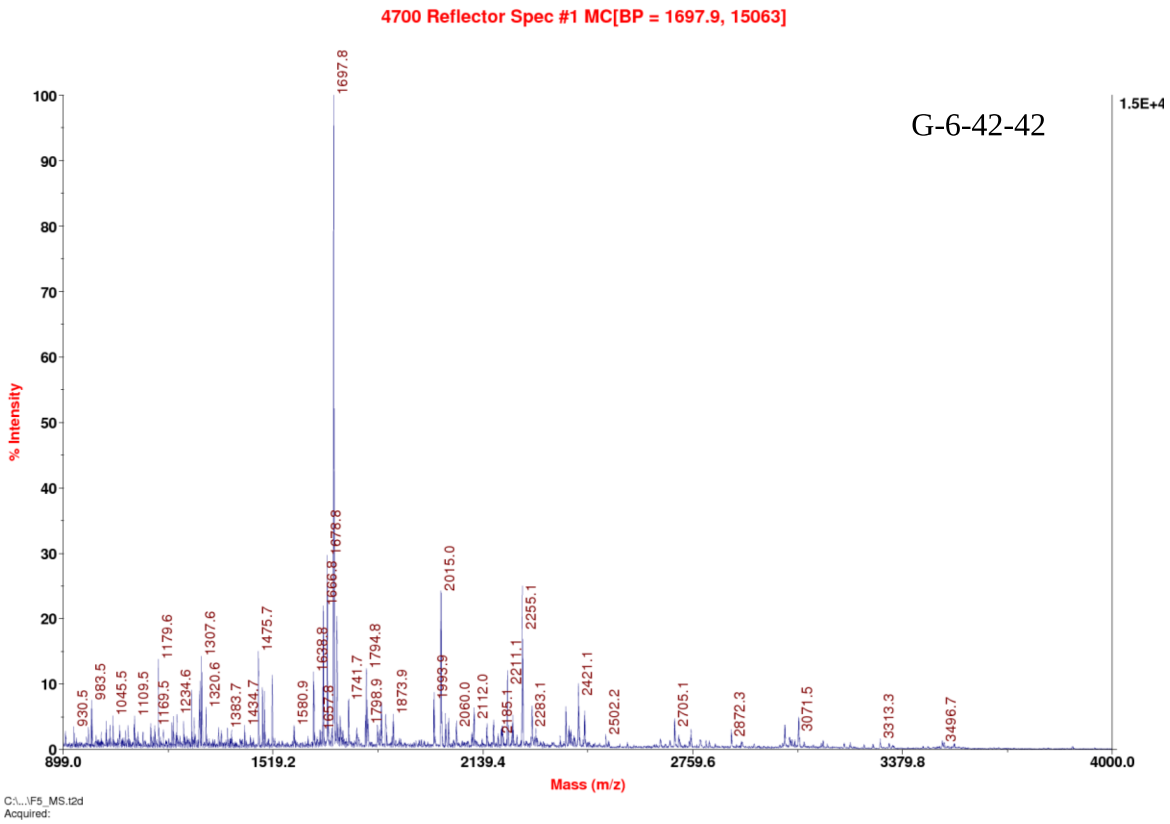
4700 Reflector Spec #1 MC[BP = 1697.9, 15063]
G-6-42-42
1.5E+4
% Intensity
Mass (m/z)
899.0
1519.2
2139.4
2759.6
3379.8
4000.0
1697.8
1678.8
1666.8
1638.8
1657.8
2015.0
2255.1
2211.1
1179.6
1307.6
1475.7
1794.8
2421.1
930.5
983.5
1045.5
1109.5
1169.5
1234.6
1320.6
1383.7
1434.7
1580.9
1741.7
1798.9
1873.9
1993.9
2060.0
2112.0
2185.1
2283.1
2502.2
2705.1
2872.3
3071.5
3313.3
3496.7
C:\...\F5_MS.t2d
Acquired:

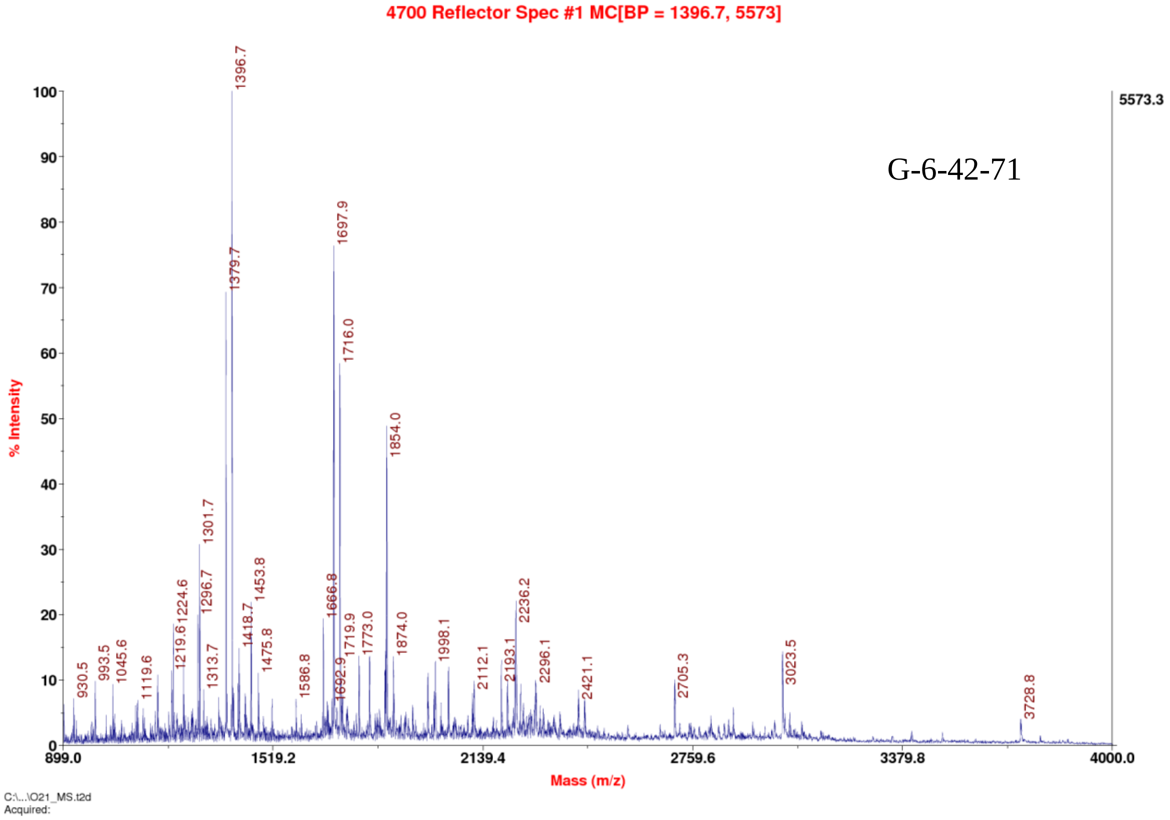
4700 Reflector Spec #1 MC[BP = 1396.7, 5573]
G-6-42-71
5573.3
% Intensity
Mass (m/z)
899.0
1519.2
2139.4
2759.6
3379.8
4000.0
1396.7
1379.7
1697.9
1716.0
1854.0
1301.7
1224.6
1219.6
1296.7
1453.8
1418.7
1475.8
1666.8
1692.9
1719.9
1773.0
1874.0
1998.1
2236.2
2193.1
2296.1
2112.1
2421.1
2705.3
3023.5
3728.8
930.5
993.5
1045.6
1119.6
1313.7
1586.8
C:\...\O21_MS.t2d
Acquired:

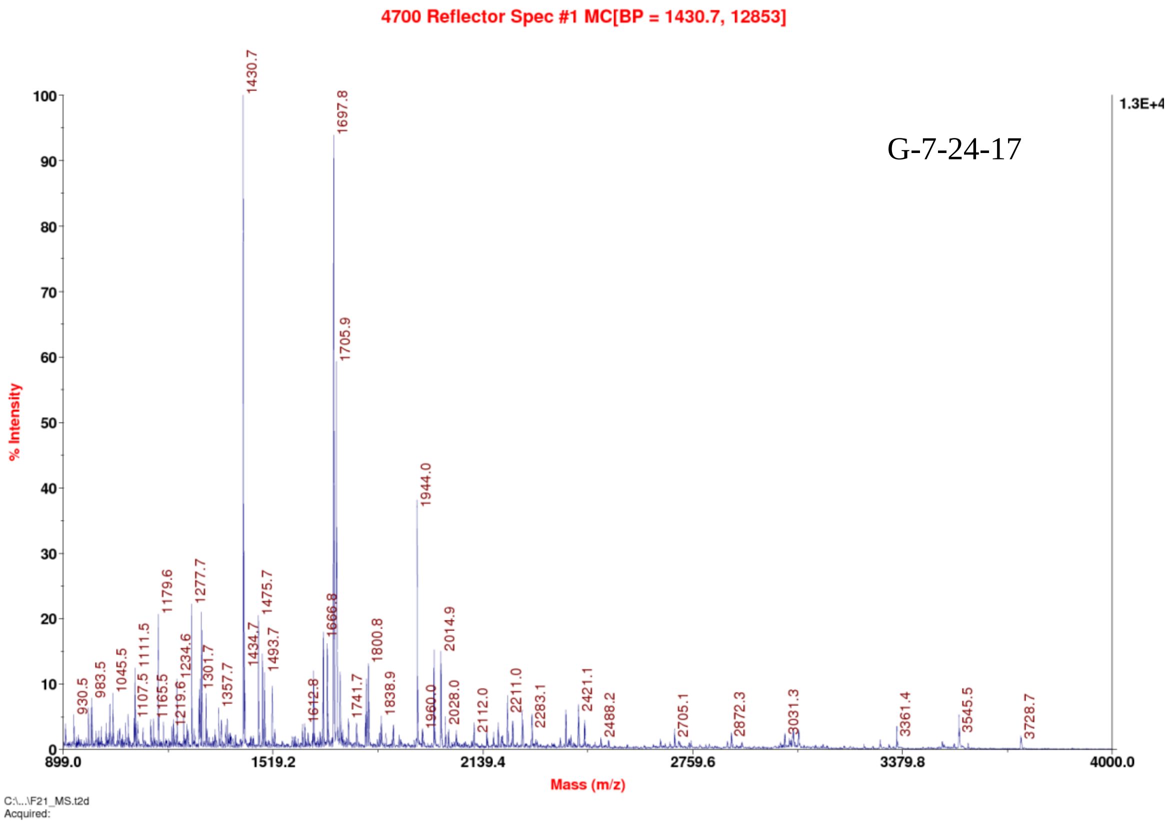
4700 Reflector Spec #1 MC[BP = 1430.7, 12853]
G-7-24-17
% Intensity
Mass (m/z)
1.3E+4
C:\...\F21_MS.t2d
Acquired:

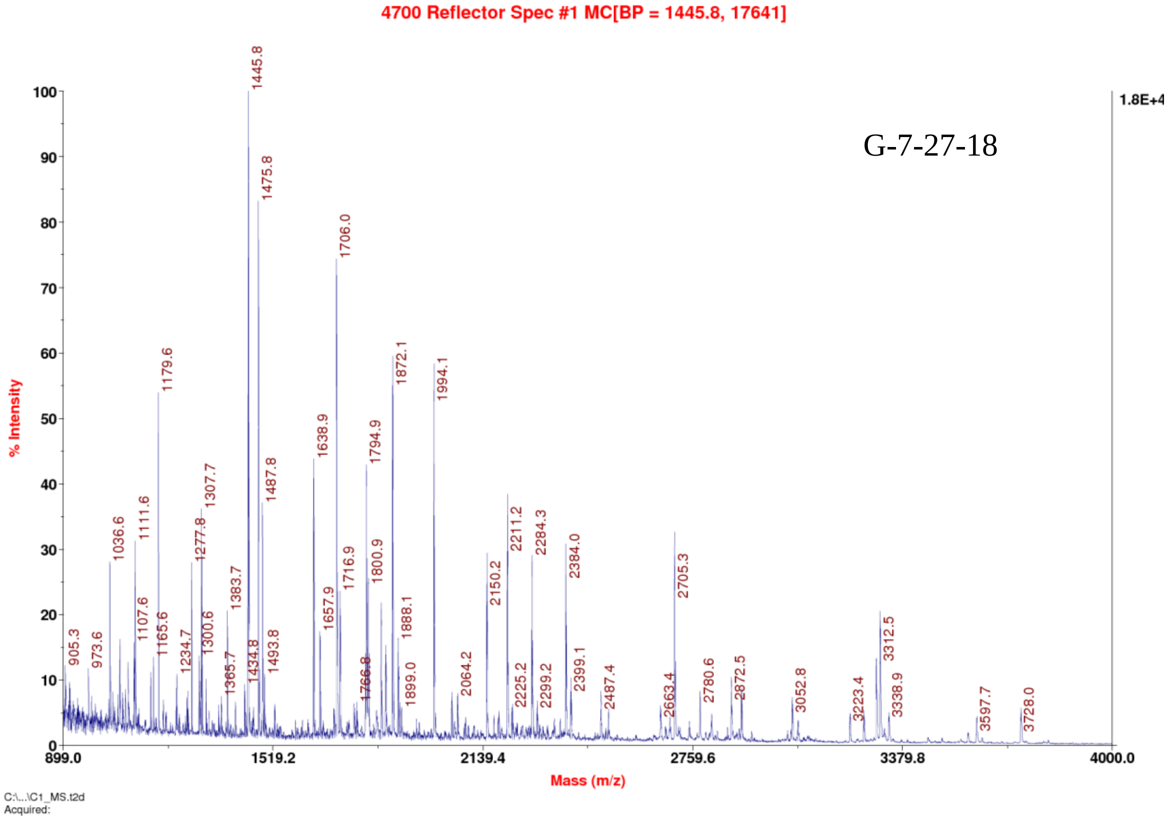
4700 Reflector Spec #1 MC[BP = 1445.8, 17641]
G-7-27-18
% Intensity
Mass (m/z)
1.8E+4
C:\...\C1_MS.t2d
Acquired:
