# Supplementary material for: Production of highly and broad-range specific monoclonal antibodies against hemagglutinin of H5-subtype avian influenza viruses and their differentiation by mass spectrometry
Source: Virol J. 2018 Jan 15;15:13. doi: 10.1186/s12985-017-0886-2 (PMC5769215; doi:10.1186/s12985-017-0886-2)
Supplement: Supplementary file 6 — Data from the advanced immunoreactivity studies. Figure S7. The ELISA titration curves of the mAbs against rHA - A/H5N1/Qinghai from a mammalian expression system. Figure S8. The ELISA titration curves of the mAbs against rHA - A/H5N1/Poland from a baculovirus expression system. Figure S9. The ELISA titration curves of the mAbs against rHA - A/H5N1/Poland from a bacterial expression system. Table S11. The concentration values interpolated from the mAb titration curves. Figure S10. Diversity of the mAb reactivities with recombinant H5 hemagglutinin proteins and H5N3 avian influenza virus. Figure S11. Relative reactivity of the mAbs with recombinant H5 hemagglutinin antigens. Figure S12. Relative reactivity of the mAbs with avian influenza viruses. (PDF 422 kb) [file 12985_2017_886_MOESM6_ESM.pdf]

Additional file 6: Data from the advanced immunoreactivity studies

The advanced immunoreactivity studies of the newly established, affinity-purified monoclonal antibodies (mAbs) were performed by ELISAs using the HA antigens described in Additional file 1: Tables S2 and S4. The non-glycosylated HA protein from a bacterial expression system (bacterial HA) was also used for testing. The protein (aa 17-522, ΔRRRKKR) with the HA sequence of the A/swan/Poland/305-135V08/2006(H5N1) AIV strain was expressed in *Escherichia coli*, refolded and purified from inclusion bodies. It was produced at the Institute of Biotechnology and Antibiotics (Warsaw, Poland). Antigenicity studies showed that our bacterial HA is recognized by commercial anti-H5 HA antibodies, listed in Additional file 1: Table S1. To distinguish between recombinant HA proteins originated from various expression systems, the rHA and rHA1 proteins produced in mammalian cells are further denoted with a "*" symbol and the rHA proteins produced in insect and bacteria cells with "**" and "***" symbols, respectively. The ELISA tests were performed following the general scheme described in the Methods. The advanced studies of the mAbs comprised the binding capability assessment and the immunoreactivity profiling.

Binding capabilities

The capability of the G-1-31-22, G-2-14-10, G-5-32-5, G-6-42-42, G-6-42-71, G-7-24-17 and G-7-27-18 mAbs to react with H5 HA antigens was assayed in an indirect ELISA by titrating them against the rHA* - A/H5N1/Qinghai immunogen. Also, the titrations against rHA** and rHA*** (A/H5N1/Poland) proteins with high homologies to the immunogen were performed. The results are shown in Figures S7-S9. Based on these results, the 4-parametric titration curves and interpolated concentration values for individual mAbs were determined. The interpolated values are presented in Table S11.

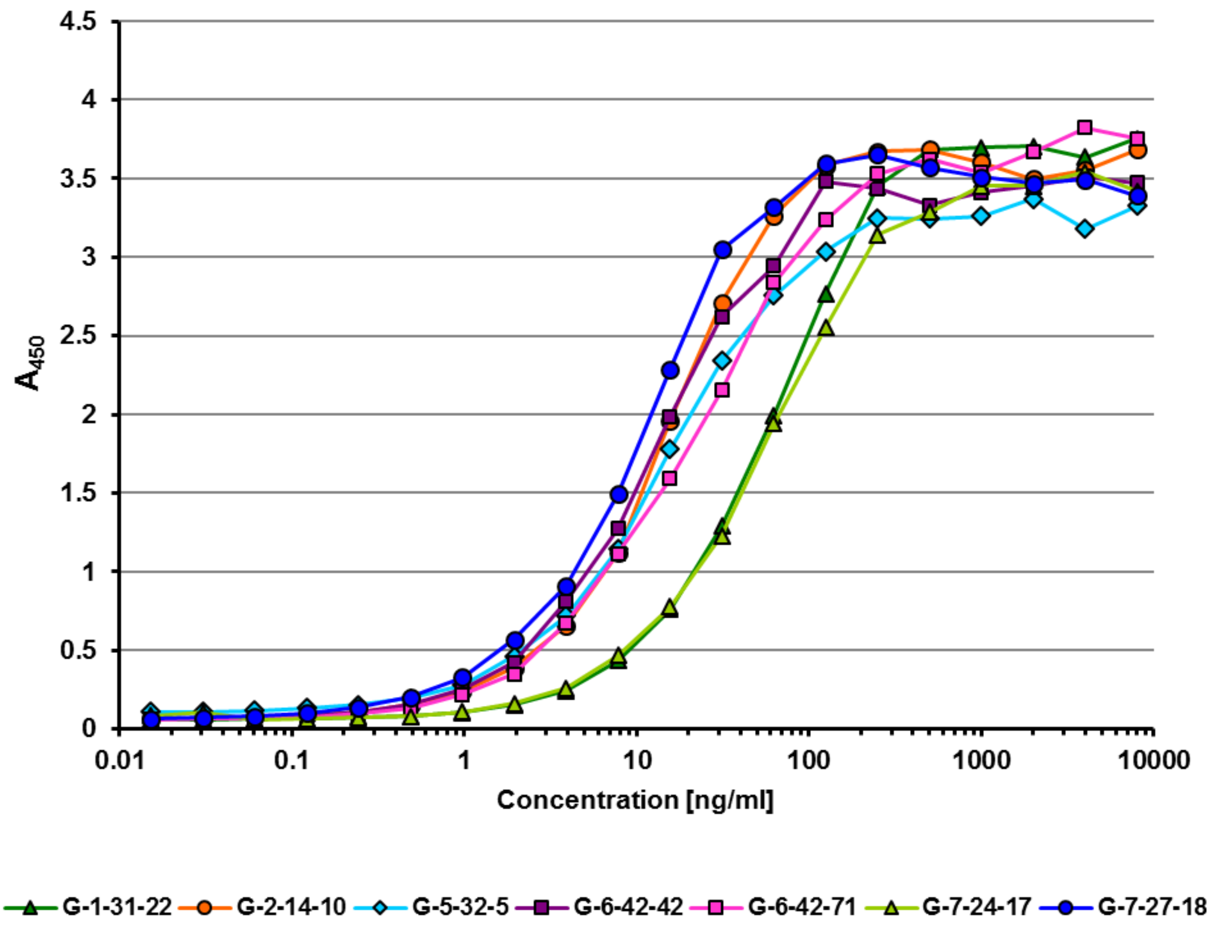

Figure S7. The ELISA titration curves of the mAbs against rHA - A/H5N1/Qinghai from a mammalian expression system.

The mAbs were tested in 2-fold serial dilutions (8 µg/mL - 0.015 ng/mL in 2% BSA/PBS) on MediSorp plates (Nunc) coated with rHA* - A/H5N1/Qinghai at 1 µg/mL in PBS. To control for non-specific binding, the mAbs at a concentration of 8 µg/mL were analyzed in the non-coated wells. The blank control was the dilution buffer. The mean absorbance values for blank control samples were subtracted.

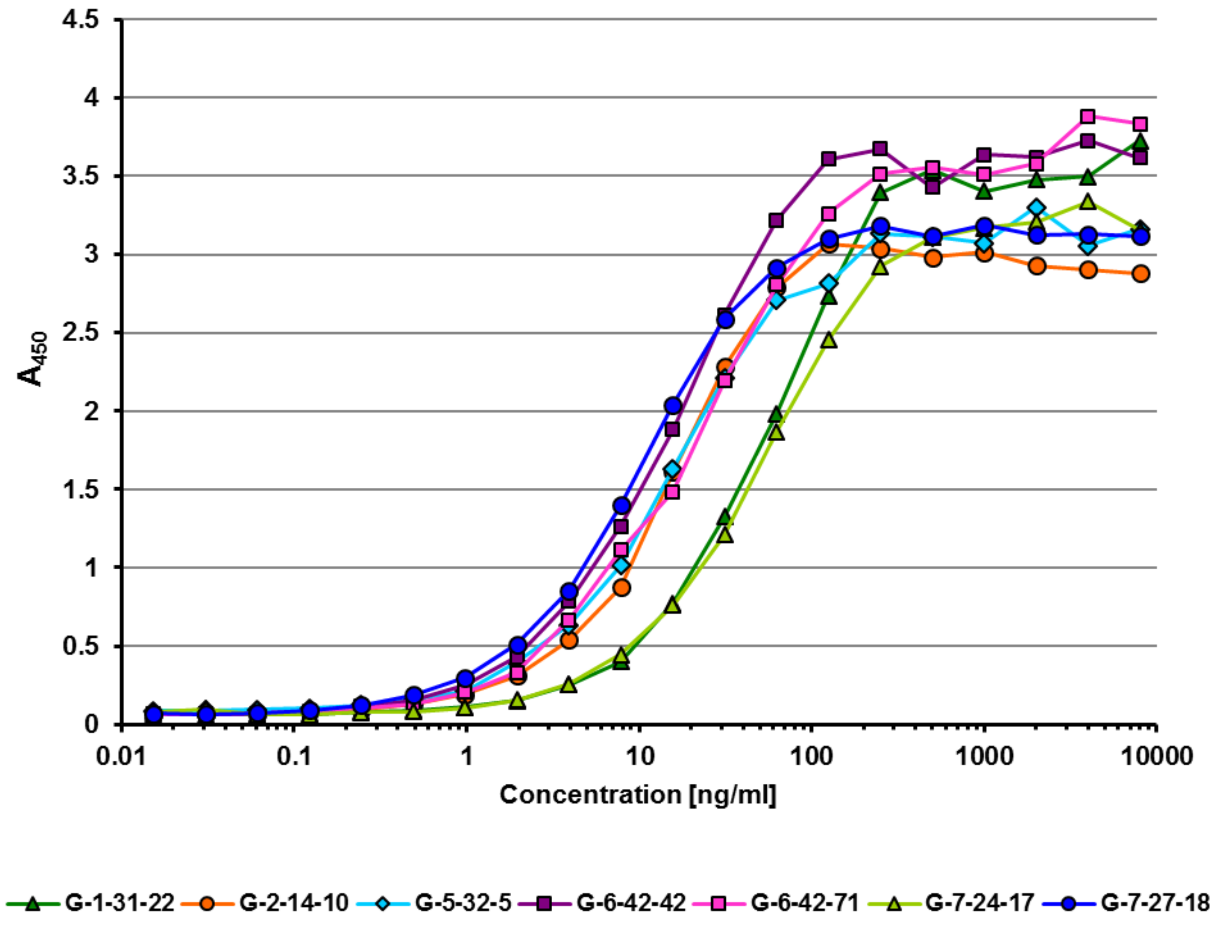

Figure S8. The ELISA titration curves of the mAbs against rHA - A/H5N1/Poland from a baculovirus expression system.

The mAbs were tested in 2-fold serial dilutions (8 µg/mL - 0.015 ng/mL in 2% BSA/PBS) on MediSorp plates (Nunc) coated with rHA** - A/H5N1/Poland at 1 µg/mL in PBS. To control for non-specific binding, the mAbs at a concentrations of 8 µg/mL were analyzed in the non-coated wells. The blank control was the dilution buffer. The mean absorbance values for blank control samples were subtracted.

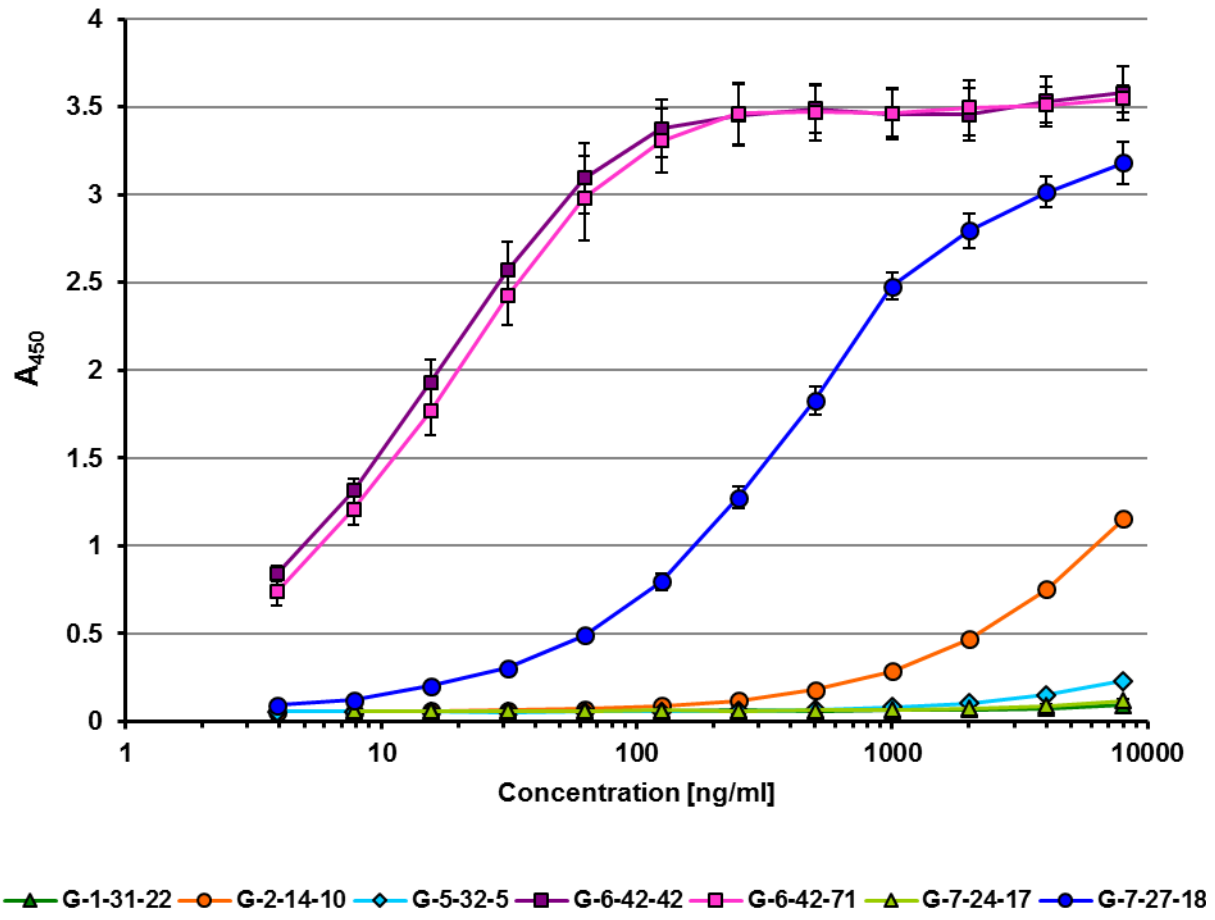

Figure S9. The ELISA titration curves of the mAbs against rHA - A/H5N1/Poland from a bacterial expression system.

The mAbs were tested in 2-fold serial dilutions (8 µg/mL - 7.8 or 3.9 ng/mL in 2% BSA/PBS) on PolySorp, MediSorp, MaxiSorp and MultiSorp plates (Nunc) coated with rHA*** - A/H5N1/Poland preparation, ~80% pure, which contained the refolded hemagglutinin at ~1 µg/mL in PBS. To control for non-specific binding, the mAbs at a concentration of 8 µg/mL were analyzed in the non-coated wells. The blank control was the dilution buffer. For each antibody sample, the mean absorbance value for blank control samples was subtracted. The results are shown as the mean A_{450} values ± SD obtained using the plates of particular types.

Table S11. The concentration values interpolated from the mAb titration curves.

mAb clones	mAb concentration for A_{450} = 1.5 [ng/mL]			mAb concentration for A_{450} = 1.0 [ng/mL]
	rHA* A/H5N1/Qinghai	rHA** A/H5N1/Poland	rHA*** A/H5N1/Poland	rHA*** A/H5N1/Poland
G-1-31-22	39	38	-	-
G-2-14-10	11	15	-	6368 ± 157
G-5-32-5	13	14	-	-
G-6-42-42	9	10	10 ± 1	5 ± 1
G-6-42-71	14	14	12 ± 2	6 ± 1
G-7-24-17	39	43	-	-
G-7-27-18	7	9	322 ± 28	161 ± 12

The titrations against rHA* (A/H5N1/Qinghai), rHA** and rHA*** (A/H5N1/Poland) proteins were performed as described in Figures S7, S8 and S9, respectively. Antibody concentrations giving the indicated signal (A_{450}) levels were determined by interpolation from the linear range of the 4-parametric titration curves using the Gene5 program (Bio-Tek). The results for rHA*** - A/H5N1/Poland are shown as means from the concentration values (± SD) interpolated from the titration curves obtained using PolySorp, MediSorp, MaxiSorp and MultiSorp plates (Nunc).

The data presented in Figures S7 and S8 and Table S11 indicate that all of the generated mAbs have the high capability of binding to the glycosylated HA proteins from mammalian and baculovirus expression systems. In contrast, only 4 out of the 7 antibody clones showed significant reactivity with non-glycosylated bacterial HA (Figure S9 and Table S11).

Immunoreactivity profiles

To facilitate comparison between the 7 antibody clones, the immunoreactivity profiles of the G -1-31-22, G-2-14-10, G-5-32-5, G-6-42-42, G-6-42-71, G-7-24-17 and G-7-27-18 mAbs were created. In Figure S10, the selected results from testing the binding of the mAbs with rHA* (A/H5N1/Qinghai), rHA** and rHA*** (A/H5N1/Poland) proteins (presented in full in Figures S7-S9), as well as with the H5N3 AIV were compiled to show some differences between the antibody clones. Further differentiation of the mAbs was accomplished by expression their reactivities with various rHA*, rHA** and rHA1* proteins and H5-subtype influenza viruses in relation to those, which were determined with rHA* - A/H5N1/Qinghai immunogen and H5N3 AIV, respectively. Thus obtained relative reactivity values are presented in Figures S11 and S12.

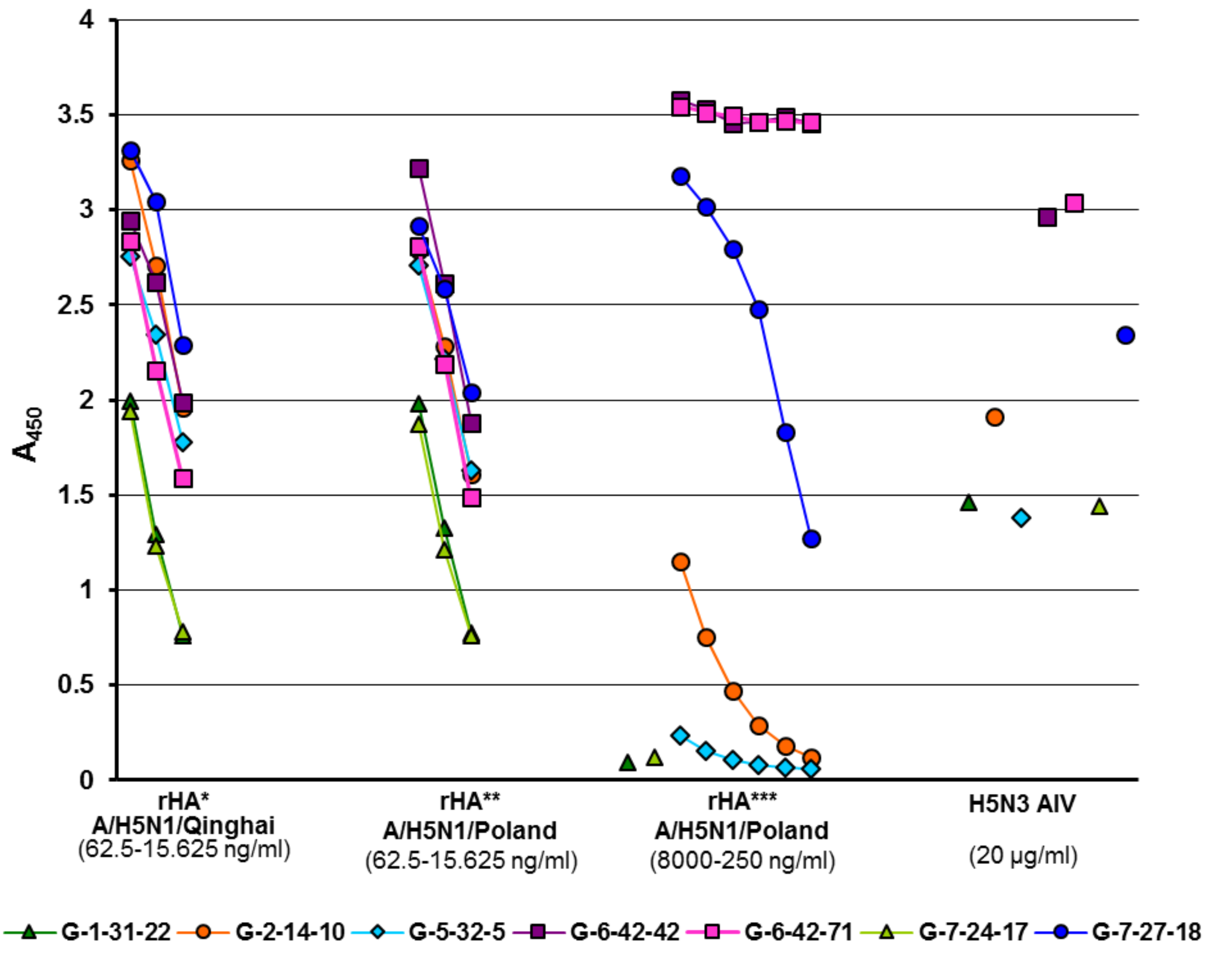

Figure S10. Diversity of the mAb reactivities with recombinant H5 hemagglutinin proteins and H5N3 avian influenza virus.

The rHA* - A/H5N1/Qinghai and rHA** - A/H5N1/Poland proteins were coated on MediSorp plates (Nunc) at 1 µg/mL in PBS. The rHA*** - A/H5N1/Poland preparation, ~80% pure, containing the refolded hemagglutinin at ~1 µg/mL in PBS, was coated on PolySorp, MediSorp, MaxiSorp and MultiSorp plates (Nunc). The H5N3 AIV was coated on MaxiSorp plate (Nunc) at 4000 hemagglutination units/mL in PBS. The mAbs were analyzed at the indicated concentrations in the antigen-coated wells and also in the non-coated wells to control for non-specific binding. In the assays with H5N3 AIV, commercial antibodies against H5 HA (mAb 8 in Additional file 1: Table S1) served as a positive control. The blank control was the dilution buffer. The mean absorbance values for blank control samples were subtracted.

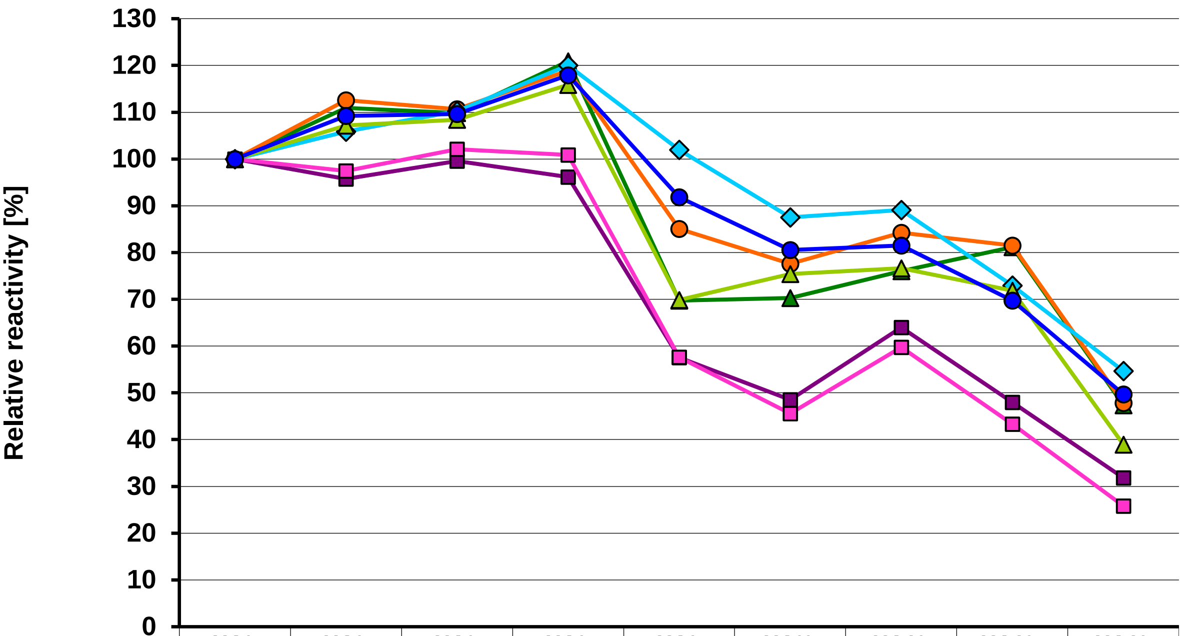

	rHA* - A/H5N1/ Qinghai	rHA* - A/H5N1/ India	rHA* - A/H5N1/ Vietnam	rHA* - A/H5N1/ Guiyang	rHA* - A/H5N2/ California	rHA** - A/H5N1/ Poland	rHA1* - A/H5N1/ Vietnam	rHA1* - A/H5N1/ HK/156	rHA1* - A/H5N1/ HK/483
G-1-31-22	100	111	110	121	70	70	76	81	47
G-2-14-10	100	113	111	119	85	78	84	81	48
G-5-32-5	100	106	110	120	102	87	89	73	55
G-6-42-42	100	96	100	96	58	49	64	48	32
G-6-42-71	100	97	102	101	58	46	60	43	26
G-7-24-17	100	107	108	116	70	75	77	72	39
G-7-27-18	100	109	110	118	92	81	82	70	50

Figure S11. Relative reactivity of the mAbs with recombinant H5 hemagglutinin antigens.

The rHA*, rHA** and rHA1* proteins were coated on Ni-NTA strips (Qiagen) at 1 μg/mL in 1% BSA/PBS. The mAbs were analyzed at concentrations from the linear range of the titrations curves against rHA* - A/H5N1/Vietnam giving the signals (A_{450}) of ~2.5. Accordingly, the G-1-31-22, G-2-14-10, G-5-32-5, G-6-42-42, G-6-42-71, G-7-24-17, G-7-27-18 mAbs were used at concentrations of 50.0, 22.5, 20.0, 8.75, 12.5, 50.0 and 12.5 ng/mL in 2% BSA/PBS, respectively. To control for non-specific binding, the mAbs were analyzed additionally in the non-coated wells. Commercial antibodies against H5 HA (mAb 8 in Additional file 1: Table S1) were used as a positive control. The blank control was the dilution buffer. The mean absorbance values for blank control samples were subtracted. The signals determined for each antibody clone against individual HA proteins were expressed as a percentage of the values obtained against rHA* - A/H5N1/Qinghai (Y-axis).

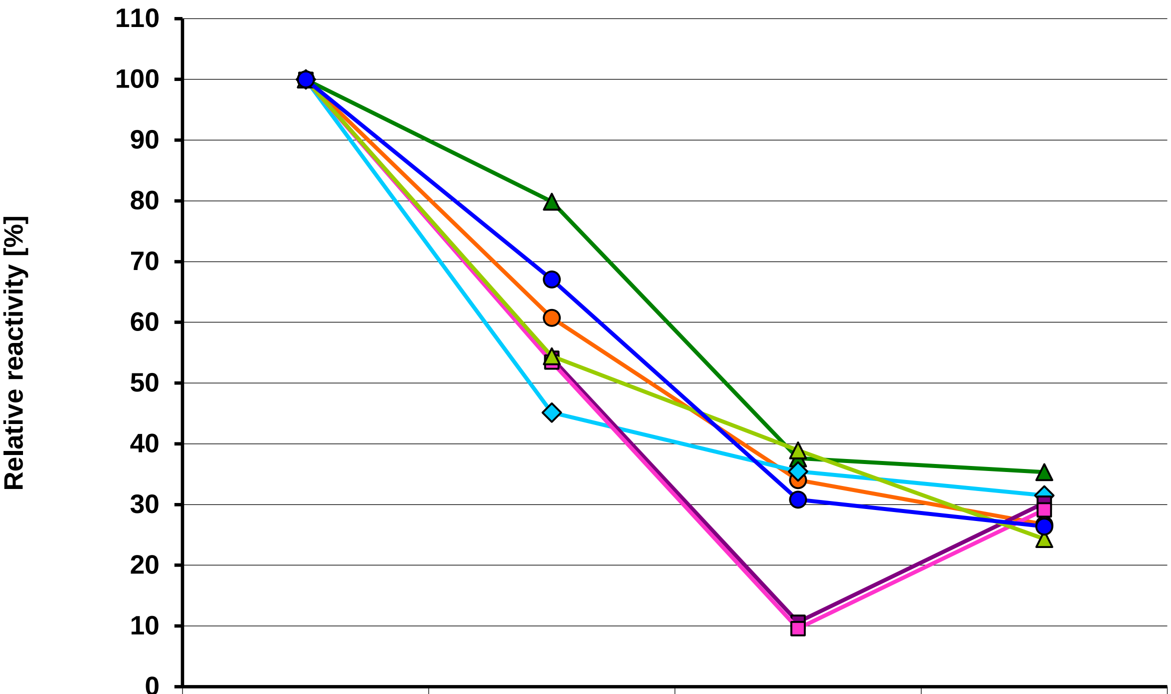

	IV - H5N3	IV - H5N1	IV - H5N9	IV- H5N2
G-1-31-22	100	80	38	35
G-2-14-10	100	61	34	27
G-5-32-5	100	45	35	31
G-6-42-42	100	54	11	30
G-6-42-71	100	53	10	29
G-7-24-17	100	54	39	24
G-7-27-18	100	67	31	26

Figure S12. Relative reactivity of the mAbs with avian influenza viruses.
The H5N1, H5N2, H5N3 and H5N9 AIVs were coated on MaxiSorp plates (Nunc) at 4000 hemagglutination units/mL in PBS. The mAbs were analyzed at 20 μg/mL in 2% BSA/PBS in the antigen-coated wells and also in the non-coated wells to control for non-specific binding. Commercial antibodies against H5 HA (mAb 8 in Additional file 1: Table S1) were used as a positive control. The blank control was the dilution buffer. The mean absorbance values for blank control samples were subtracted. The signals (A_{450}) determined for individual antibody clones against each of the H5N1, H5N9 and H5N2 viruses were expressed as a percentage of the values obtained against the H5N3 AIV (Y-axis).

A close inspection of the data presented in Figures S10-S12 enables to conclude that the analyzed antibodies differ in their immunoreactivity profiles. Accordingly, 6 different clones could be distinguished among the 7 selected mAbs.
